# 2-Methoxyestradiol Protects Against Lung Ischemia/Reperfusion Injury by Upregulating Annexin A1 Protein Expression

**DOI:** 10.3389/fimmu.2021.596376

**Published:** 2021-03-16

**Authors:** Wen-I Liao, Shu-Yu Wu, Shih-Hung Tsai, Hsin-Ping Pao, Kun-Lun Huang, Shi-Jye Chu

**Affiliations:** ^1^The Graduate Institute of Medical Sciences, National Defense Medical Center, Taipei, Taiwan; ^2^Department of Emergency Medicine, Tri-Service General Hospital, National Defense Medical Center, Taipei, Taiwan; ^3^Institute of Aerospace and Undersea Medicine, National Defense Medical Center, Taipei, Taiwan; ^4^Department of Physiology and Biophysics, Graduate Institute of Physiology, National Defense Medical Center, Taipei, Taiwan; ^5^Department of Internal Medicine, Tri-Service General Hospital, National Defense Medical Center, Taipei, Taiwan

**Keywords:** 2-methoxyestradiol, acute lung injury, ischemia and reperfusion, epithelium, annexin A1

## Abstract

**Background:** 2-Methoxyestradiol (2ME), a natural 17-β estradiol metabolite, is a potent anti-inflammatory agent, but its effect on ischemia/reperfusion (IR)-induced acute lung inflammation remains unknown. Annexin A1 (AnxA1), a glucocorticoid-regulated protein, is effective at inhibiting neutrophil transendothelial migration by binding the formyl peptide receptors (FPRs). We aimed to investigate whether 2ME upregulates the expression of AnxA1 and protects against IR-induced lung damage.

**Methods:** IR-mediated acute lung inflammation was induced by ischemia for 40 min followed by reperfusion for 60 min in an isolated, perfused rat lung model. The rat lungs were randomly treated with vehicle or 2ME, and the functional relevance of AnxA1 was determined using an anti-AnxA1 antibody or BOC2 (a pan-receptor antagonist of the FPR). *In vitro*, human primary alveolar epithelial cells (HPAECs) and rat neutrophils were pretreated with 2ME and an AnxA1 siRNA or anti-AnxA1 antibody and subjected to hypoxia-reoxygenation (HR).

**Results:** 2ME significantly decreased all lung edema parameters, neutrophil infiltration, oxidative stress, proinflammatory cytokine production, lung cell apoptosis, tight junction protein disruption, and lung tissue injury in the IR-induced acute lung inflammation model. 2ME also increased the expression of the AnxA1 mRNA and protein and suppressed the activation of nuclear factor-κB (NF-κB). *In vitro*, 2ME attenuated HR-triggered NF-κB activation and interleukin-8 production in HPAECs, decreased transendothelial migration, tumor necrosis factor-α production, and increased apoptosis in neutrophils exposed to HR. These protective effects of 2ME were significantly abrogated by BOC2, the anti-AnxA1 antibody, or AnxA1 siRNA.

**Conclusions:** 2ME ameliorates IR-induced acute lung inflammation by increasing AnxA1 expression. Based on these results, 2ME may be a promising agent for attenuating IR-induced lung injury.

## Introduction

Acute lung injury (ALI)/acute respiratory distress syndrome (ARDS) is a serious illness characterized by severe pulmonary edema and a profound inflammatory response in the lung. The mortality rate of patients with severe ARDS is as high as 46%, and the disease has a poor prognosis (1). Life-threatening acute lung inflammation can be induced by diverse ischemia/reperfusion (IR) injury conditions, including resuscitation for cardiac arrest, cardiopulmonary bypass, hemorrhagic shock, pulmonary embolism, and lung transplantation (2). Restoration of blood flow after ischemia is frequently associated with harmful effects that are characterized by epithelial and endothelial dysfunction, neutrophil infiltration, inflammatory cytokine release, and further recruitment of more neutrophils and macrophages into the alveoli (3). The exacerbation of inflammation initiated by lipid peroxidation and oxygen free radicals results in increased microvascular permeability and ultimately leads to acute lung edema (3). Currently, effective drugs are not available for treating IR-induced acute lung inflammation in the clinic, and the available treatment strategies are limited to supportive care (4).

2-Methoxyestradiol (2ME) is a natural endogenous metabolite of 17-β estradiol with low binding affinity for estrogen receptors and antitumor, antiangiogenic, and antiproliferative effects (5). In addition, 2ME has been shown to exert protective effects on several inflammation-associated diseases, such as rheumatoid arthritis and experimental autoimmune encephalomyelitis (6, 7). 2ME ameliorates multiple organ injury in mice with cecal ligation and puncture-induced sepsis by attenuating inflammatory cytokine production (8). 2ME reduces antigen-induced airway remodeling in a murine model of ovalbumin-induced inflammatory pulmonary disease (9). Furthermore, 2ME has been shown to protect against kidney and brain IR injury by reducing nuclear factor-κB (NF-κB) activity (10, 11).

The annexin A1 (AnxA1) protein is an important glucocorticoid-regulated endogenous inhibitor of inflammation that is expressed at high levels in lung, brain, kidney, and heart tissues, as well as various cells, such as neutrophils, fibroblasts, macrophages, and epithelial cells (12). AnxA1 exerts anti-inflammatory effect by interacting with formyl peptide receptors (FPRs) and reduces vascular inflammatory responses associated with IR injury (13, 14). Upon binding to the FPRs on neutrophils, AnxA1 limits neutrophil adhesion to the endothelium and transmigration, decreasing the production of proinflammatory mediators in the alveolar space (15). We recently reported a novel role for the exogenous AnxA1 N-terminal peptide in ameliorating IR-induced acute lung inflammation in rats by inhibiting the NF-κB pathway, and the protective effect was abrogated by BOC2 (a pan FPR antagonist) (16).

Currently, researchers have not clearly determined whether 2ME attenuates IR-induced acute lung inflammation. Studies have demonstrated that 17β-estradiol exhibited anti-inflammatory effects through upregulating expression of AnxA1 protein (17, 18). Since 2ME is a biologically active metabolite of 17β-estradiol, 2ME may exhibit anti-inflammatory effects through modulating the AnxA1 expression. Besides, 2ME has been shown similar anti-inflammatory properties to a glucocorticoid (dexamethasone) in acute lung inflammation (19). Further, AnxA1 is a glucocorticoid-regulated protein that mediates many of the anti-inflammatory effects of glucocorticoids (20). Based on these observations, we aimed to test whether 2ME would regulate, at least partially, AnxA1 protein expression that mediates the anti-inflammatory properties of 2ME. In our preliminary study, 2ME significantly increased the expression of endogenous AnxA1 in lung tissues (Supplementary Figure 3A), which may be related to the protective mechanism of 2ME. Although a possible protective role for AnxA1 has been extensively described in several models of IR injury, the modulation of this anti-inflammatory protein after the administration of pharmacological strategies, such as 2ME, remains unknown. Therefore, in this study, we investigated whether 2ME decreased IR-induced acute lung inflammation by upregulating the expression of endogenous AnxA1.

## Materials and Methods

### Animals

All experiments were approved by the Institutional Animal Care and Use Committee of the National Defense Medical Center (approval number: IACUC-15-077, 19-March-2015). The care of male Sprague-Dawley rats (350 ± 20 g) was provided in accordance with the Guide for the Care and Use of Laboratory Animals. Rats were housed in a temperature-controlled room with a 12-h light-dark cycle.

### Isolated Perfused Rat Lung Model

Briefly, the rats were ventilated with humidified 21% O_2_ containing 5% CO_2_ at a 1-cm H_2_O positive end-expiratory pressure via a tracheostomy, and body temperature was maintained at 37°C with heating pads. The ventilator had a respiratory rate of 60 breaths per min and delivered a tidal volume of 3 ml. A median sternotomy was performed, the right ventricle was injected with 1 U of heparin/g body weight (BW), and 10 ml of blood was collected by cardiac puncture. Cardiac arrest developed immediately after the cardiac puncture. Then, a cannula was inserted into the pulmonary artery, and another wide-bore cannula was placed in the left ventricle and advanced into the left atrium through the mitral valve. The pulmonary venous pressure (PVP) and pulmonary artery pressure (PAP) were continuously recorded from the side arm of the cannula. The isolated lung was perfused with a “half-blood” perfusate containing 10 ml of a physiological salt solution (PSS) and a 10-ml sample of collected blood. The PSS contained 119 mM NaCl, 4.7 mM KCl, 1.17 mM MgSO_4_, 22.6 mM NaHCO_3_, 1.18 mM KH_2_PO_4_, 1.6 mM CaCl_2_, 5.5 mM glucose, 50 mM sucrose, and 4% bovine serum albumin. The flow rate was constantly maintained at a rate of 8 ml/min by a roller pump. The real-time changes in the lung weight (LW) were recorded by an electronic balance, upon which the *in situ* isolated perfused lung was placed (21).

### Design of the *in vivo* Experiment

We used the anti-AnxA1 antibody (Santa Cruz Biotechnology, USA) and N-Boc-Phe-Leu-Phe-Leu-Phe (BOC2, a non-selective FPR antagonist, ICN Pharmaceuticals, UK) to inhibit the action of AnxA1 and investigate whether AnxA1 was involved in the protective effects of 2ME. The neutralizing properties of the anti-AnxA1 antibody (Santa Cruz Biotechnology) were examined using immunoprecipitation and neutrophil transmigration assay (Supplementary Figure 6). The isolated rat lungs were randomly assigned to the control + dimethyl sulfoxide (DMSO), control + 2ME (20 mg/kg BW, intraperitoneal injection (i.p.), Sigma-Aldrich, USA), control + anti-AnxA1 antibody (60 μg/kg BW, i.p., Santa Cruz Biotechnology), IR + DMSO, IR + different dosages of 2ME (5, 10, 20 mg/kg BW), IR + anti-AnxA1 antibody, IR + BOC2 (50 μg per rat, i.p.), IR + anti-AnxA1 antibody + 2ME (20 mg/kg BW), or IR + BOC2 + 2ME (20 mg/kg BW) group (*n* = 6 rats per group). 2ME was dissolved in 0.5% DMSO in saline and injected intraperitoneally 60 min prior to IR. Rats were pretreated with the anti-AnxA1 antibody or BOC2 for 30 min before the 2ME injection in the IR + anti-AnxA1 antibody + 2ME or IR + BOC2 + 2ME group or prior to IR in the IR + anti-AnxA1 antibody, and IR + BOC2 groups. The doses of 2ME and BOC2 used in this study were determined based on previous studies and our preliminary investigations (10, 16) (Supplementary Figure 3). The dose of anti-AnxA1 antibody used in the present study (60 μg/kg BW) was selected according to the dose in our *in vitro* study that inhibited rat neutrophil migration (1 μg/ml). Lung ischemia was induced in rats in the IR group by stopping ventilation and perfusion for 40 min and then restoring them to allow reperfusion for another 60 min. The rats were ventilated with humidified 21% O_2_ containing 5% CO_2_ when ventilation and perfusion were restored after 40 min of ischemia. The PaCO_2_, PaO_2_, and pH levels in the perfusate were measured by an ABL 800FLEX blood gas analyzer (Radiometer, Denmark) before the start of ischemia and after the restoration of reperfusion for 60 min. The diagrams of the experimental design *in vivo* and *in vitro* are provided in Supplementary Figures 1, 2, respectively.

### Vascular Filtration Coefficient

The vascular filtration coefficient (K_f_) was calculated from the elevation in the venous pressure-induced LW change, as previously described (22, 23). K_f_ was defined as the *y*-intercept of the plot (in g·min^−1^) divided by the PVP (10 cm H_2_O) and LW and reported in whole units of g·min^−1^ cm H_2_O^−1^ × 100 g.

### LW/BW and Wet/Dry (W/D) Weight Ratios

The right middle lobe was removed at the hilar region, weighed on an electronic balance, and recorded as the wet LW after the experiments. The LW/BW ratios were determined as the wet LW divided by BW. For the dry weight, the middle portion of the right lung was dried for 48 h at 60°C in an oven, and the W/D weight ratios were calculated.

### Assessment of Protein Concentrations and Interleukin (IL)-6, Cytokine-Induced Neutrophil Chemoattractant (CINC)-1, Tumor Necrosis Factor (TNF)-α, and IL-8 Levels in Bronchoalveolar Lavage Fluid (BALF) and in Culture Supernatants

The BALF was obtained by rinsing the left lung with 2.5 ml of phosphate-buffered saline (PBS) twice and then centrifuging the sample at 200*g* for 10 min. A bicinchoninic acid test was used to measure the protein level in the supernatant. IL-6, CINC-1, and TNF-α levels in BALF and in rat neutrophil culture supernatants, and IL-8 levels in human primary alveolar epithelial cells (HPAECs) culture supernatants were quantified using commercial rat and human ELISA kits (R&D Systems, USA) according to the manufacturer's instructions.

### Immunohistochemistry

Immunostaining for AnxA1, myeloperoxidase (MPO), CD45, Ly-6C, Ly-6G, Gal-3, FPR1, FPR2, and cleaved caspase-3 was performed on 5-μm-thick sections of rat lung tissue. Briefly, formalin-fixed paraffin lung tissue sections were deparaffinized before antigen retrieval. The endogenous peroxidases in lung tissue were quenched with 3% H_2_O_2_ and 100% methanol for 15 min. The sections were immunostained with a rabbit polyclonal antibody against AnxA1 (1:100, Bioss, USA), a rabbit polyclonal antibody against MPO (1:200, Thermo Fisher Scientific, USA), a mouse monoclonal antibody against CD45 (1:100, Merck KGaA, Germany), a mouse monoclonal antibody against Ly6C (1:200, Santa Cruz Biotechnology), a rabbit polyclonal antibody against Ly6G (1:300, Biorbyt, UK), a mouse monoclonal antibody against Gal-3 (1:100, Santa Cruz Biotechnology), and a rabbit polyclonal anti-cleaved caspase-3 antibody (1:200, CST, USA). Sections were washed twice with PBS containing 0.1% Tween-20 (PBST) and then incubated with a rat-specific horseradish peroxidase (HRP)-conjugated secondary antibody for 30 min. The HRP signal was visualized after a chromogenic reaction with diaminobenzidine for 5 min, and the lung tissue sections were counterstained with hematoxylin. Subsequently, the slides were dehydrated in a gradient of alcohol solutions (16).

### Detection of the Protein Carbonyl Content and Malondialdehyde (MDA) Level in Lung Tissues

The methods for measuring the MDA level and protein carbonyl content were described previously (16). The protein carbonyl contents and MDA levels in the upper lobe of the right lung were determined using a Protein Carbonyl Content Assay Kit (Abcam, USA) and an MDA Assay Kit (Abcam), respectively, according to the manufacturer's instructions. The results of both assays are reported as nmol/mg protein.

### Real-Time Quantitative PCR

Total RNA extraction and cDNA synthesis were performed using a Direct-zol RNA MiniPrep Kit (Zymo Research, USA) and MMLV Reverse Transcription Kit (Protech, Taiwan), respectively, according to the manufacturer's instructions. Probes (AnxA1: Rn00563742_m1, FPR1: Rn01441684_s1, FPR2: Rn03037051_gH, and Actb: Rn00667869_m1) were purchased from Thermo Fisher Scientific. Thereafter, real-time PCR was performed using an Eco Real-Time PCR system (Illumina, USA) and 2X qPCRBIO Probe Blue Mix Lo-ROX (PCR Biosystems, USA). The real-time PCR mixture contained 1 μl of 20X TaqMan Gene Expression Assay, 10 μl of 2X qPCRBIO Probe Blue Mix, and 1 μl of cDNA templates in a total volume of 20 μl. The PCR cycling conditions were an initial incubation at 95°C for 2 min followed by 45 cycles of denaturation at 95°C for 5 s and annealing and extension at 60°C for 20 s.

### Western Blotting

Lung tissues and protein lysates from cultured cells (30 μg/lane) were electrophoresed via 8–12% sodium dodecyl sulfate-polyacrylamide gel electrophoresis and transferred onto a polyvinylidene fluoride membrane. The membranes were blocked with 5% milk overnight to prevent non-specific binding. The blots were probed with one of the following primary antibodies: anti-AnxA1 (1:1,000, Bioss), anti-lamin B1 (1:100, Santa Cruz Biotechnology), anti-Bcl-2, anti-NF-κB p65, anti-phospho-NF-κB p65, anti-IκB-α, anti-IκB kinase (IKK)-β, anti-phospho-IKK-α/β, anti-ERK1/2, anti-phosho-ERK1/2, anti-p38, anti-phospho-p38 (1:1,000, CST), or β-actin (1:10,000, Sigma Chemical Company, USA). The blots were then washed with PBST for 10 min and incubated with HRP-conjugated goat anti-rabbit IgG (1:20,000) or goat anti-mouse IgG (1:20,000) secondary antibodies at room temperature for 1 h. After three washes with PBST for 10 min, the immunoreactive bands were visualized with an enhanced chemiluminescence kit and exposed using a UVP ChemiDoc-It Imaging System. The density of the immunoblots was calculated using image analysis software. The level of each protein was measured by repeating the Western blot analysis at least three times. The data are presented as the relative ratio of the target protein to the reference protein. The relative ratio of the target protein in the control group was arbitrarily set to 1.

### Immunofluorescence Staining

Immunofluorescence staining was performed as previously described (16). Briefly, the primary antibodies rabbit polyclonal anti-claudin-3 and anti-occludin, mouse monoclonal anti-ZO-1 (1:200; Invitrogen, USA), rabbit polyclonal anti-AnxA1 (1:100, Bioss), rabbit polyclonal anti-Ly6G (1:200, Biorbyt), rabbit polyclonal anti-FPR-1(1:100, Santa Cruz Biotechnology), mouse monoclonal anti-FPR-2 (1:100, Santa Cruz Biotechnology), and rabbit polyclonal anti-cleaved-caspase-3 (1:400, CST) were used for immunofluorescence labeling. The ZO-1-labeled sections were incubated with a goat anti-mouse IgG-fluorescein isothiocyanate-labeled secondary antibody (green, diluted 1:200; Santa Cruz Biotechnology) for 30 min at room temperature. DyLight 633-labeled goat anti-rabbit IgG (red, diluted 1:200; Invitrogen) was used as the secondary antibody for the claudin-3 and occludin antibodies. Images were acquired using a fluorescence microscope (Leica DM 2500, Wetzlar, Germany).

### Histopathological Analysis

Lung tissues were fixed with 10% formalin, embedded in paraffin, sliced into 4-μm sections, and stained with hematoxylin and eosin (H&E). Ten high-power fields at 400× magnification were used to count the numbers of polymorphonuclear leukocytes (PMN) and assign a lung injury score to each lung tissue sample. A light microscope (Olympus CKX41, Japan) was used to examine the morphological features. A minimum of 10 randomly selected fields was inspected for neutrophil infiltration in the airspace or vessel wall and thickening of the alveolar wall. A four-point scale was used to define the lung section score (16). In this classification, no (0), mild (1), moderate (2), or severe (3) lung injury was examined by two pathologists in a blinded manner. The two scores for each sample were summed for a total score ranging from 0 to 6 that represented the lung injury score.

### Cell Viability Assays

Cell viability was determined using Premix WST-1 Cell Proliferation Reagent (Cayman Chemical, USA). HPAECs and rat neutrophils were plated in 96-well plates at a density of 6,000 cells per well and incubated overnight. 2ME was added at concentrations of 0, 0.1, 1, 10, and 100 μM or 0, 0.5, 1, 2, and 5 μM into three 96-well plates containing HPAECs or rat neutrophils, respectively. The HPAECs or rat neutrophils were then incubated with 10 μl of WST-1 per well for 0, 0.5, 1, 2, and 24 h or 0, 1, and 2 h, respectively. The final results were measured with a plate reader at 450 nm.

### Cell Culture and Induction of HR

HPAECs (BCRC Cat# H6053) obtained from Cell Biologics were isolated from normal human lung tissue. Cells were routinely grown on plastic with Epithelial Cell Medium Kits (BCRC Cat# H6621) in a humidified atmosphere of 5% CO_2_-95% room air. Neutrophils from adult male Sprague-Dawley rats (250–350 g) were freshly isolated using a previously described method (24). Before performing functional tests, neutrophils were allowed to recover for 30 min at 37°C in Roswell Park Memorial Institute (RPMI) 1640 medium (Gibco, USA) supplemented with 10% fetal bovine serum (FBS) (Thermo Fisher Scientific). Cells were resuspended in incubation buffer prior to functional assays. For AnxA1 knockdown, an AnxA1 small interfering RNA (siRNA) and empty vectors were purchased from Dharmacon (Lafayette, USA). The vectors were transfected into HPAECs with Lipofectamine RNAiMax (Thermo Fisher Scientific). The transfection efficiency was assessed using fluorescence microscopy and Western blot analysis. For hypoxia/reoxygenation (HR) induction, HPAECs or rat neutrophils were subjected to 24 or 2 h of hypoxia (1% O_2_, 5% CO_2_, and 94% N_2_), respectively, followed by 1 h of reoxygenation (5% CO_2_ and 95% room air) at 37°C. Before HR induction, HPAECs or rat neutrophils were pretreated with 2ME (1 μM) or 2ME (2 μM) and the anti-AnxA1 antibody (1 μg/ml), respectively. Normal mouse IgG (Santa Cruz Biotechnology) was used as the antibody control. Treatment conditions for rat neutrophils comprised (1) DMSO + IgG, (2) 2ME + IgG, and (3) 2ME + anti-AnxA1 antibody groups. The control group was maintained in the reoxygenated state without a hypoxic stimulus.

### Rat Neutrophil Transmigration Assay

Neutrophil chemotaxis was evaluated using a Transwell system (3-μm pore size, Corning Costar, USA). First, 500 μl of RPMI 1640 medium containing 10% FBS were added to the lower well of the migration plate before the neutrophils were added. Then, 1.5 × 10^6^ freshly isolated neutrophils in 150 μl of serum-free RPMI 1640 medium were added to the upper chamber. The agents, 2 μM 2ME and anti-AnxA1 antibody, were then added directly to the cell suspension in the upper chamber. After exposure to hypoxia for 2 h and reoxygenation for 1 h, neutrophils were harvested from the lower chamber and analyzed using an ADAM MC Auto Cell Counter or by adding BCECF-AM cell staining solution. The results are presented as the percentage of cells that had migrated to the lower chamber.

### Immunocytochemistry/Immunofluorescence Staining of HPAECs and Rat Neutrophils

After exposure to hypoxia for 24 h (HPAECs) or 2 h (neutrophils) followed by reoxygenation for 1 h, the cells were fixed and stained. Neutrophils were isolated using a Cytospin column (Thermo Shandon Cytospin 3) and centrifugation at 1,200 rpm for 5 min, and then the slides were air dried for 20 min at room temperature to increase cell adhesion and reduce loss during the subsequent staining procedure. Images were acquired using a fluorescence microscope (Leica DMi8, Germany).

### Data and Statistical Analysis

Based on our previous report (23), the assumption of Cohen's *d* was defined as 3.98 [(1.097 – 0.207)/0.223607], which was decided to large effect size. Cohen's *d* was calculated by subtracting the population mean (Control group's post-reperfusion K_f_) from the sample mean (IR group's post-reperfusion K_f_). The two-tailed significant level and the expected power were set to 0.05 and 0.8, respectively. Six rats in each group were sufficient to distinguish the difference between the two groups calculated using GPower version 3.1.9.6. For the calculations of the expected animal attrition, the number per group was counted to be a maximum of eight per group, considering the potential animal loss due to possible complications during surgical interventions or anesthesia induction. All statistical calculations were performed using GraphPad Prism 6 statistical software (GraphPad Software, USA). Data are presented as the means ± standard deviation. Comparisons among groups were analyzed using one-way analysis of variance (ANOVA) followed by Bonferroni's *post-hoc* correction to evaluate the difference between each of the treatment and control groups. The increases in LW between groups were compared using two-way ANOVA followed by the Bonferroni *post-hoc* test. A value of *p* < 0.05 was defined as significant.

## Results

### 2ME Attenuated IR-Induced Lung Edema

**Figure 1:**
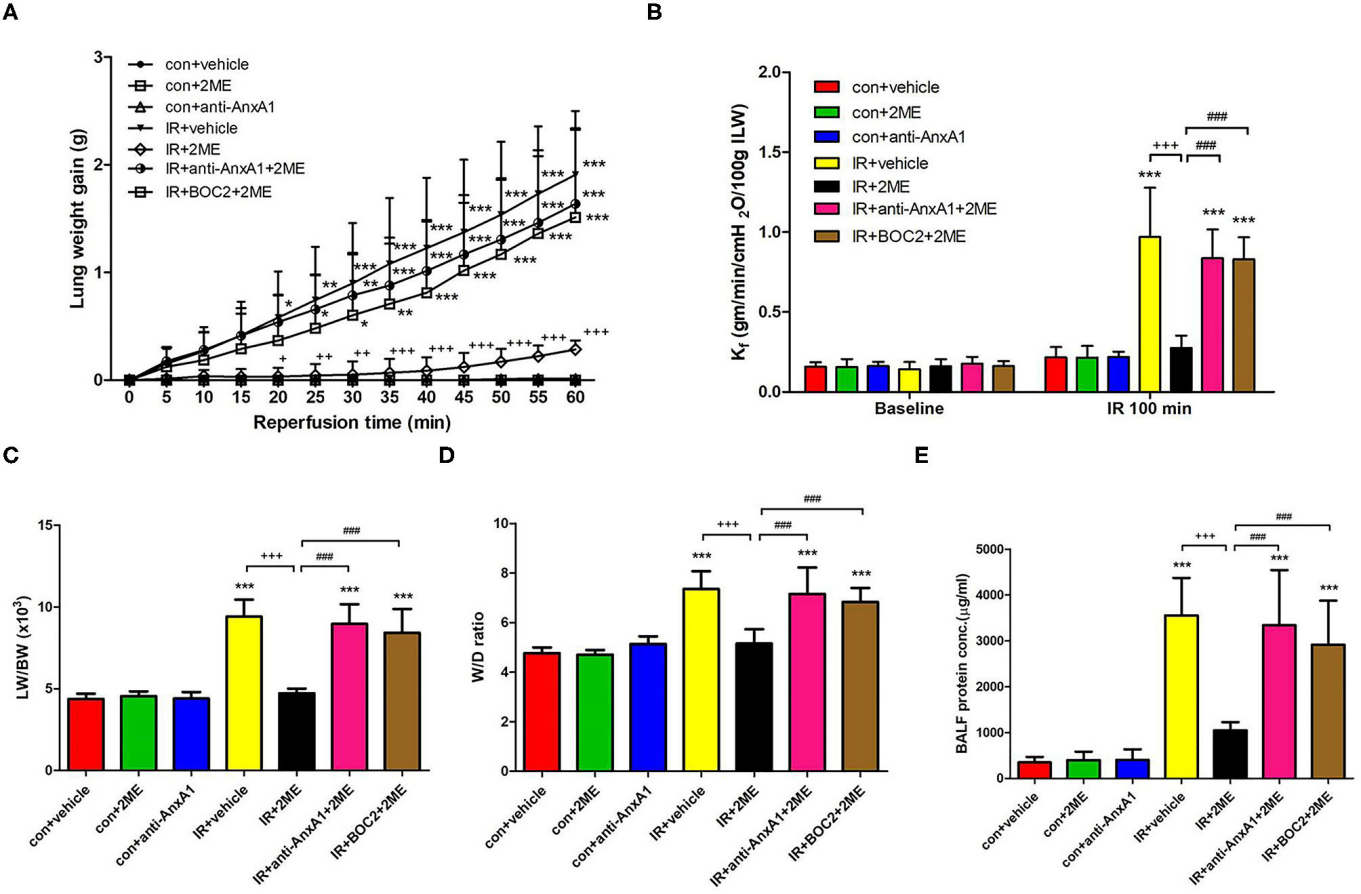
2ME alleviated IR-induced acute lung edema. **(A)** Lung weight gain, **(B)** K_f_, **(C)** LW/BW, **(D)** W/D weight ratios, and **(E)** the protein concentration in BALF increased significantly in the IR group. The increases in these parameters were significantly ameliorated by 2ME administration. The protective effect of 2ME was abrogated by the addition of the anti-AnxA1 antibody or BOC2. Data are presented as the means ± SD (*n* = 6 rats per group). ^*^p < 0.05, ^**^p < 0.01, and ^***^p < 0.001 compared with the control group; ^+^*p* < 0.05, ^++^*p* < 0.01, and ^+++^*p* < 0.001 compared with the IR group; and ^###^*p* < 0.001 compared with the IR + 2ME group.

When compared to the vehicle control, IR injury significantly increased the LW, K_f_, LW/BW ratio, W/D weight ratio, and protein concentrations in the BALF. The anti-AnxA1 antibody and BOC2 showed a tendency to aggravate IR damage, which did not reach statistical significance (not shown). Compared to rats subjected to IR injury, rats pretreated with 2ME exhibited significantly attenuated IR-induced lung edema in a dose-dependent manner (Figures 1A–E and Supplementary Figures 3B–F). However, the addition of the anti-AnxA1 antibody or BOC2 significantly blocked the protective effects of 2ME on IR-induced lung edema. There was no statistically significant difference in the perfusate pH and PaCO_2_ levels between groups. Compared with the vehicle control, IR injury significantly increased the difference between final PaO_2_ and baseline PaO_2_ levels. The 2ME pretreatment significantly reduced the PaO_2_ difference in the IR group (Supplementary Table 1).

### 2ME Increased the Expression of the AnxA1 mRNA and Protein in Lung Tissues

**Figure 2:**
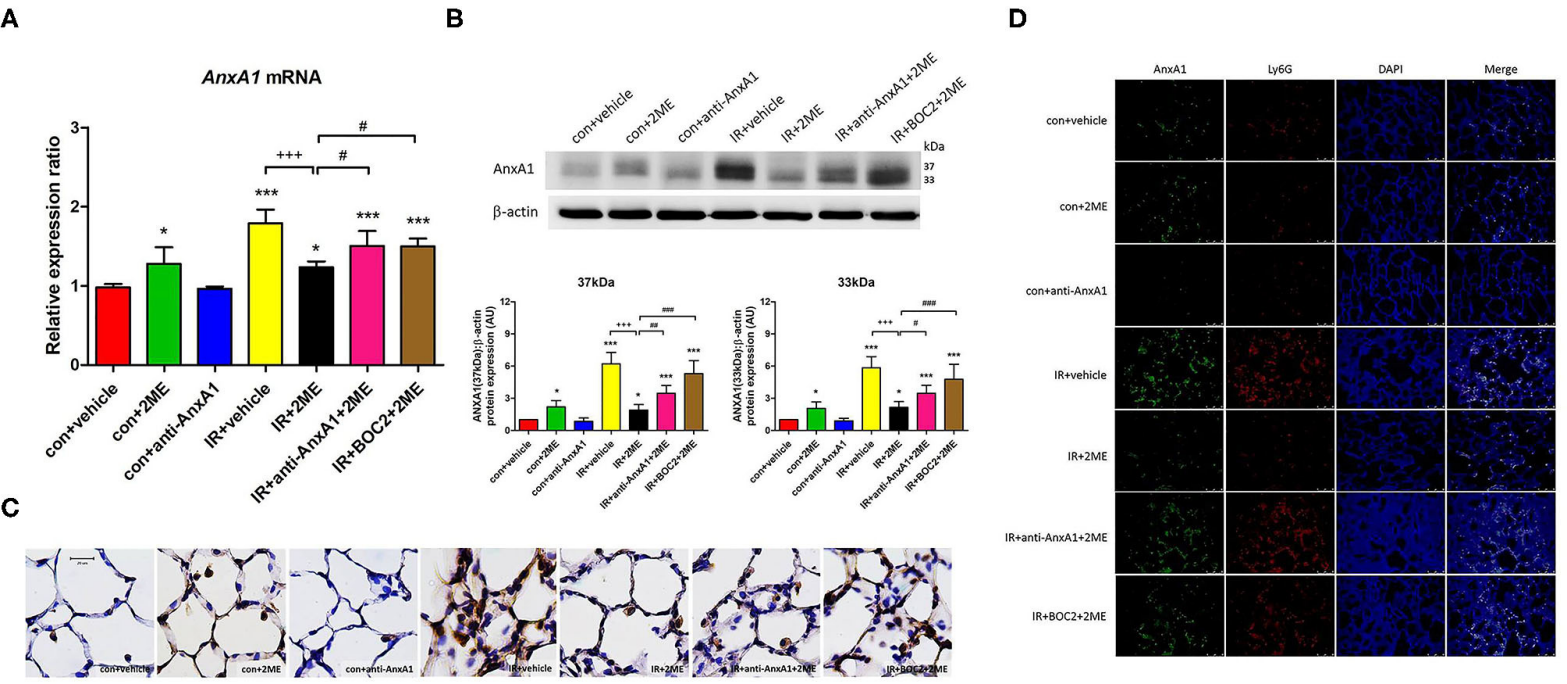
2ME increased AnxA1 mRNA and protein expression in normal lung tissues but decreased AnxA1 mRNA and protein expression in IR-injured lung tissues. Quantitative real-time PCR and Western blot analyses revealed that IR significantly increased the levels of the **(A)** AnxA1 mRNA and **(B)** both the intact and cleaved AnxA1 protein in the lung (as detected by the presence of the 37- or 33-kDa fragment) compared with the control group. Furthermore, the IR + 2ME group showed significantly decreased expression of the AnxA1 mRNA and protein in the lung compared with the IR group. Following anti-AnxA1 antibody or BOC2 pretreatment, the expression of the AnxA1 protein in the lung was significantly increased compared with the IR + 2ME group. **(C)** Serial paraffin-embedded sections of the rat lungs were immunohistochemically stained to assess AnxA1 expression (400× magnification). 2ME increased the number of AnxA1-stained epithelial cells compared with the vehicle alone in the control group. A large number of AnxA1-stained neutrophils infiltrated into the lung alveolar space upon IR injury, and the number was reduced after 2ME administration. The addition of the anti-AnxA1 antibody or BOC2 reversed the effect of 2ME. **(D)** Immunofluorescence analysis of AnxA1 protein in the lung sections after IR injury (400× magnification). Sections were co-stained for AnxA1 (green), Ly6G (red), and DAPI (blue). Images show co-localization of AnxA1 protein with Ly6G positive cells in the lung tissues of control or IR-challenged rats. ^*^p < 0.05 and ^***^p < 0.001 compared with the control group; ^+++^*p* < 0.001 compared with the IR group; and ^#^*p* < 0.05, ^##^*p* < 0.01, and ^###^*p* < 0.001 compared with the IR + 2ME group.

Western blotting and qPCR were performed to quantify the AnxA1 content in lung tissue. Under unstimulated conditions, an intraperitoneal injection of 2ME induced a significant increase in AnxA1 expression at the mRNA and protein (intact and cleaved fragments) levels in the 2ME control group. Compared with the vehicle control group, the IR group exhibited a significant increase in the expression of the AnxA1 mRNA and protein. Furthermore, compared with the IR group, the 2ME pretreatment significantly decreased the expression of the AnxA1 mRNA and protein in the 2ME + IR group (Figures 2A–D). The addition of the anti-AnxA1 antibody or BOC2 reversed the effect of 2ME on IR injury. Immunohistochemical staining for AnxA1, FPR1, and FPR2 in lung tissues also produced similar results (Figure 2C, Supplementary Figures 7A, 8A). To further determine whether increased AnxA1 expression was mainly attributed to neutrophils in IR-treated lungs, we performed double immunofluorescence staining of lung sections using antibodies for AnxA1 and the neutrophil marker Ly6G. We found that AnxA1 protein expression highly co-localized with infiltrated neutrophils in the lung sections (Figure 2D).

### 2ME Ameliorated IR-Induced Proinflammatory Cytokine Production in the BALF and Histopathological Changes

**Figure 3:**
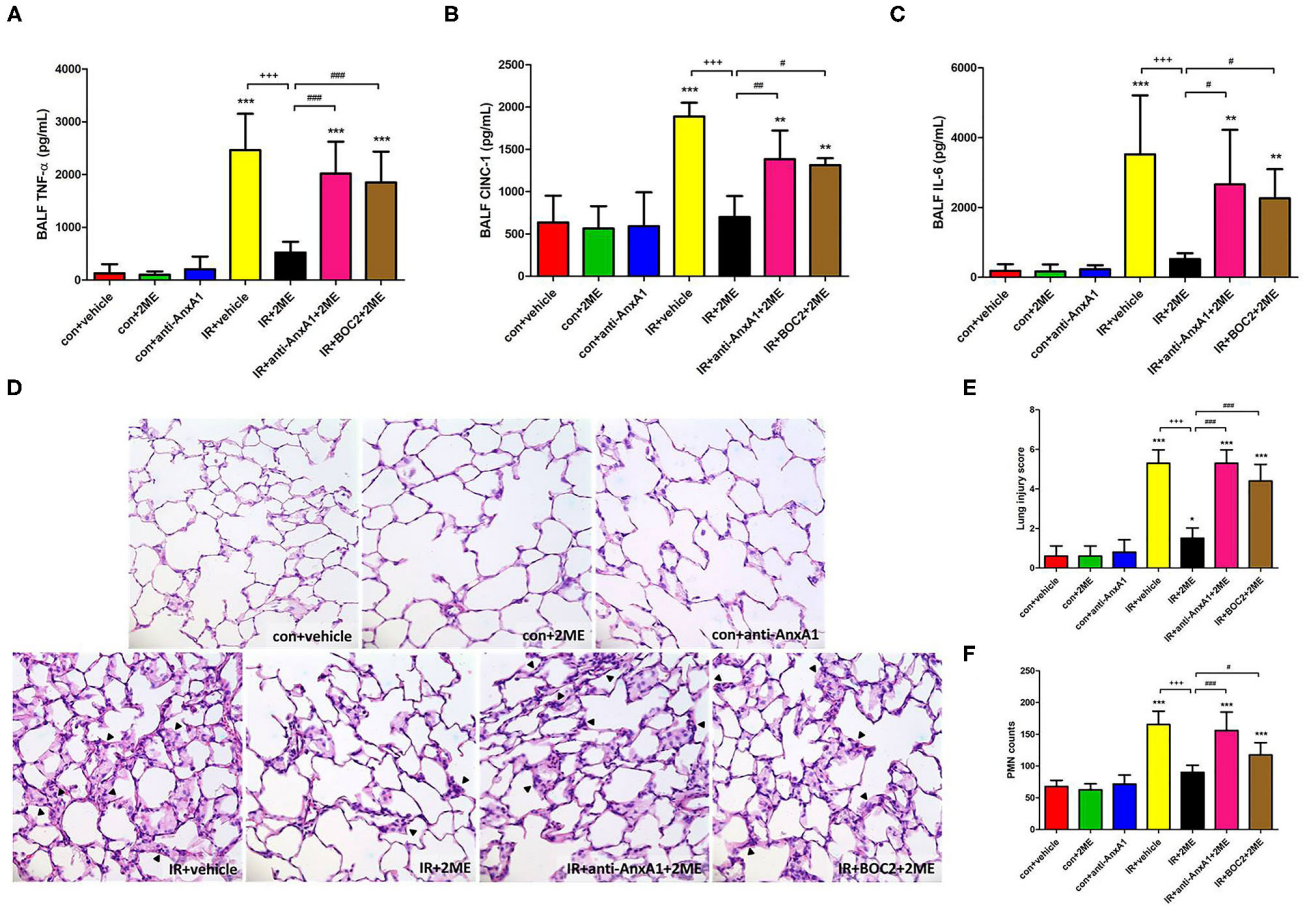
2ME reduced the IR-induced production of proinflammatory cytokines in the BALF and the severity of histological features in IR-injured lung tissue. 2ME significantly decreased IR-induced **(A)** TNF-α, **(B)** CINC-1, and **(C)** IL-6 levels in the BALF. The addition of the anti-AnxA1 antibody or BOC2 significantly abolished the anti-inflammatory effect of 2ME. **(D)** The lung tissue sections were stained with hematoxylin and eosin and examined under a light microscope (400× magnification). Lung injury was increased in the IR group, as evidenced by the increased neutrophil infiltration (arrowheads) and alveolar wall thickness. The 2ME pretreatment improved the histopathological changes. **(E)** Similar results were obtained by determining a histological lung injury score. **(F)** The numbers of infiltrated neutrophils per high power field (400× magnification) were elevated in the IR group, while the addition of 2ME significantly reduced the number of neutrophils, despite the IR injury. These improvements were all abrogated by the addition of the anti-AnxA1 antibody or BOC2. Data are presented as the means ± SD (*n* = 6 rats per group). ^*^p < 0.05, ^**^p < 0.01, and ^***^p < 0.001 compared with the control group; ^+++^*p* < 0.001 compared with the IR group; and ^#^*p* < 0.05, ^##^*p* < 0.01, and ^###^*p* < 0.001 compared with the IR + 2ME group.

Compared with the control group, the IR group displayed significantly higher levels of proinflammatory cytokines, such as IL-6, CINC-1, and TNF-α, in the BALF. 2ME administration significantly attenuated these increases. Moreover, these protective effects of 2ME were abrogated by the addition of the anti-AnxA1 antibody or BOC2 (Figures 3A–C). CD45, Ly6C, Ly6G, and Gal-3 are markers of PMNs, monocytes, neutrophils, and macrophages, respectively. Morphological observations of the control group showed a normal thickness of the lung alveolar wall and neutrophil infiltration. Obvious thickening of the alveolar walls and increased neutrophil infiltrates and CD45, Ly6C, Ly6G, and Gal-3 staining were observed in the IR group. The 2ME pretreatment significantly decreased neutrophil infiltration into the lungs, CD45, Ly6C, Ly6G, and Gal-3 staining and the histological lung injury score in the IR group. However, the administration of the anti-AnxA1 antibody or BOC2 prevented the beneficial effect of the 2ME pretreatment (Figures 3D–F and Supplementary Figure 4).

### 2ME Decreased the Protein Carbonyl Content, MDA Level, and Number of MPO-Positive Cells in Lung Tissue Exposed to IR

**Figure 4:**
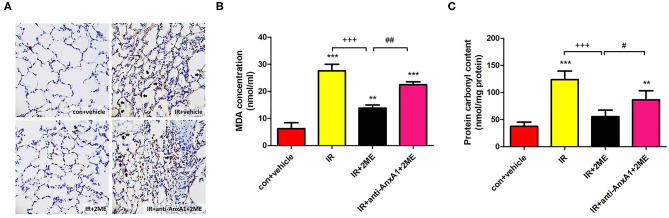
2ME decreased oxidative stress induced by IR injury in lung tissues. The number of **(A)** MPO-positive cells and the **(B)** MDA and **(C)** protein carbonyl levels in the rat lung tissues increased significantly after lung IR injury, whereas the 2ME pretreatment significantly attenuated these changes. The antioxidant effects of 2ME were blocked by the addition of the anti-AnxA1 antibody. **(A)** Immunohistochemistry for MPO in the lung (200× magnification). Black arrowheads indicate MPO-positive cells. Data are presented as the means ± SD (*n* = 6 rats per group). ^**^p < 0.01 and ^***^p < 0.001 compared with the control group; ^+++^*p* < 0.001 compared with the IR group; and ^#^*p* < 0.05 and ^##^*p* < 0.01 compared with the IR + 2ME group.

Compared to rats subjected to IR injury, rats pretreated with 2ME prior to IR injury exhibited a significant attenuation of the IR-induced increases in the number of MPO-positive cells, protein carbonyl content, and MDA level in the lung tissue (Figures 4A–C). However, these antioxidant effects of 2ME were blocked by the administration of the anti-AnxA1 antibody.

### 2ME Increased Bcl-2 Levels but Reduced the Level of the Cleaved Caspase-3 Protein in Lung Tissues Exposed to IR

**Figure 5:**
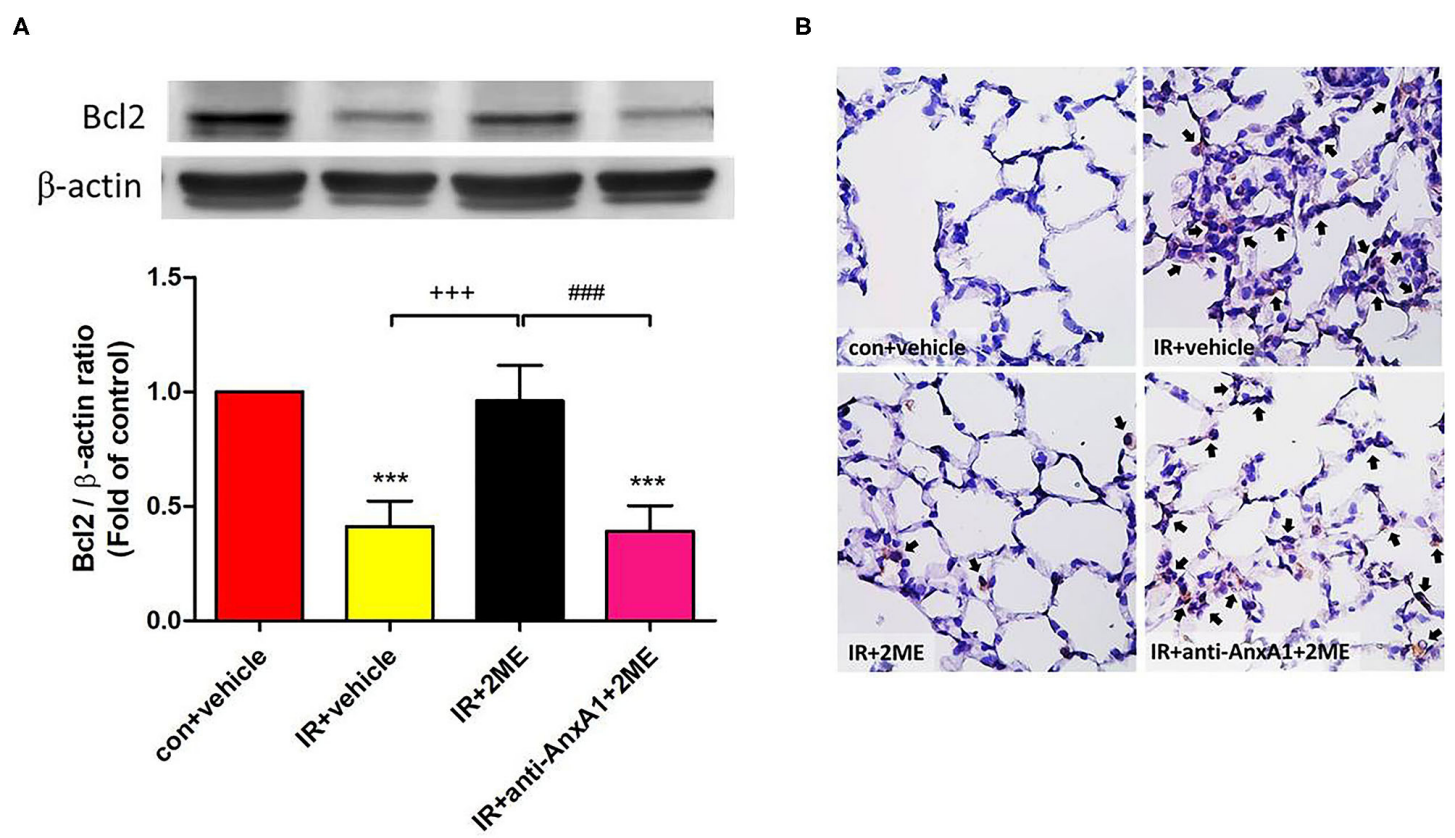
2ME attenuated IR-induced lung cell apoptosis. **(A)** Western blot showing the expression of Bcl-2 in the lung tissue. β-Actin served as a loading control for cytoplasmic proteins. Representative blots are shown. **(B)** Immunohistochemically staining (400× magnification) for cleaved caspase-3 expression in the lung tissue (indicated with black arrows). A large number of cleaved caspase-3-immunolabeled cells and significantly decreased expression of the Bcl-2 protein were observed in the IR group compared with the control group. The 2ME pretreatment significantly increased Bcl-2 protein expression and reduced the staining for cleaved caspase-3 in the IR group. Finally, when the anti-AnxA1 antibody was added, the antiapoptotic effects of 2ME on the lung tissue were abolished. ^***^p < 0.001 compared with the control group; ^+++^*p* < 0.001 compared with the IR group; and ^###^*p* < 0.001 compared with the IR + 2ME group.

Compared to the IR group, the 2ME pretreatment significantly increased the level of the Bcl-2 protein and decreased the number of activated caspase-3-immunolabeled cells in the 2ME + IR group. The administration of the anti-AnxA1 antibody reversed these effects of 2ME (Figures 5A,B).

### 2ME Restored Claudin-3, Occludin, and ZO-1 Expression in the Lung Epithelium Exposed to IR

**Figure 6:**
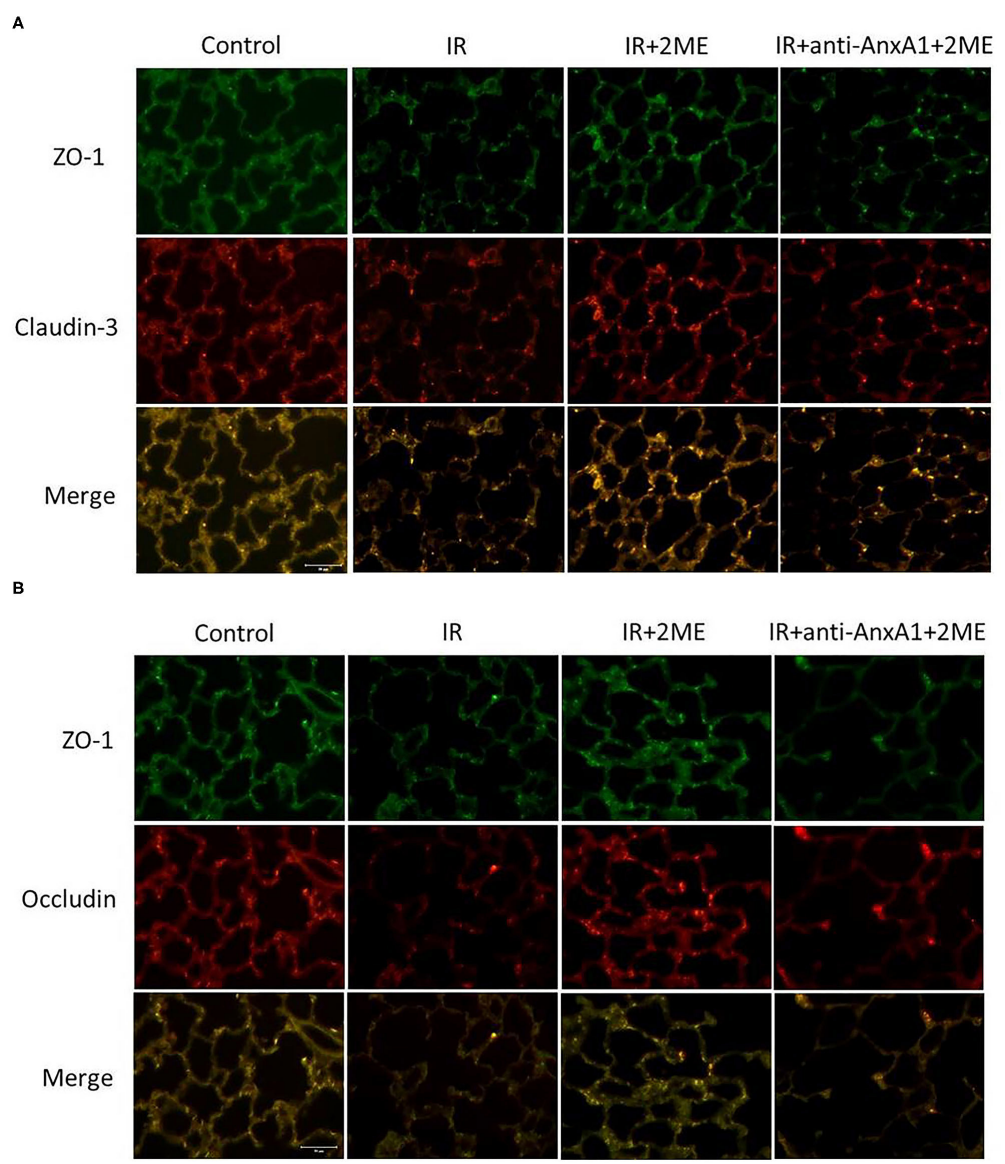
2ME suppressed IR-induced disruption of tight junction proteins. Lung sections were immunostained for claudin-3, occludin, and ZO-1 (400× magnification). The control group showed strong linear staining of tight junction proteins, including **(A)** claudin-3 (red), **(B)** occludin (red), and **(A,B)** ZO-1 (green). The IR injury group exhibited fragmented and low-intensity staining of these tight junction proteins in the alveolar septa. The 2ME pretreatment reversed the disruption of these tight junction proteins, but the protective effect of 2ME was abrogated by the addition of the anti-AnxA1 antibody.

We evaluated the expression of the claudin-3, occludin, and ZO-1 proteins in lung tissues to assess the integrity of tight junction proteins. The lung epithelium in the control group exhibited strong linear staining for claudin-3, occludin, and ZO-1. The IR group showed fragmented and low-intensity staining in the alveolar walls. The 2ME pretreatment reversed the effects of IR, but the protective effects of 2ME were abolished by the addition of the anti-AnxA1 antibody (Figures 6A,B).

### 2ME Reduced the Activation of the NF-κB and Mitogen-Activated Protein Kinase (MAPK) Signaling Pathways in Lung Tissues Exposed to IR

**Figure 7:**
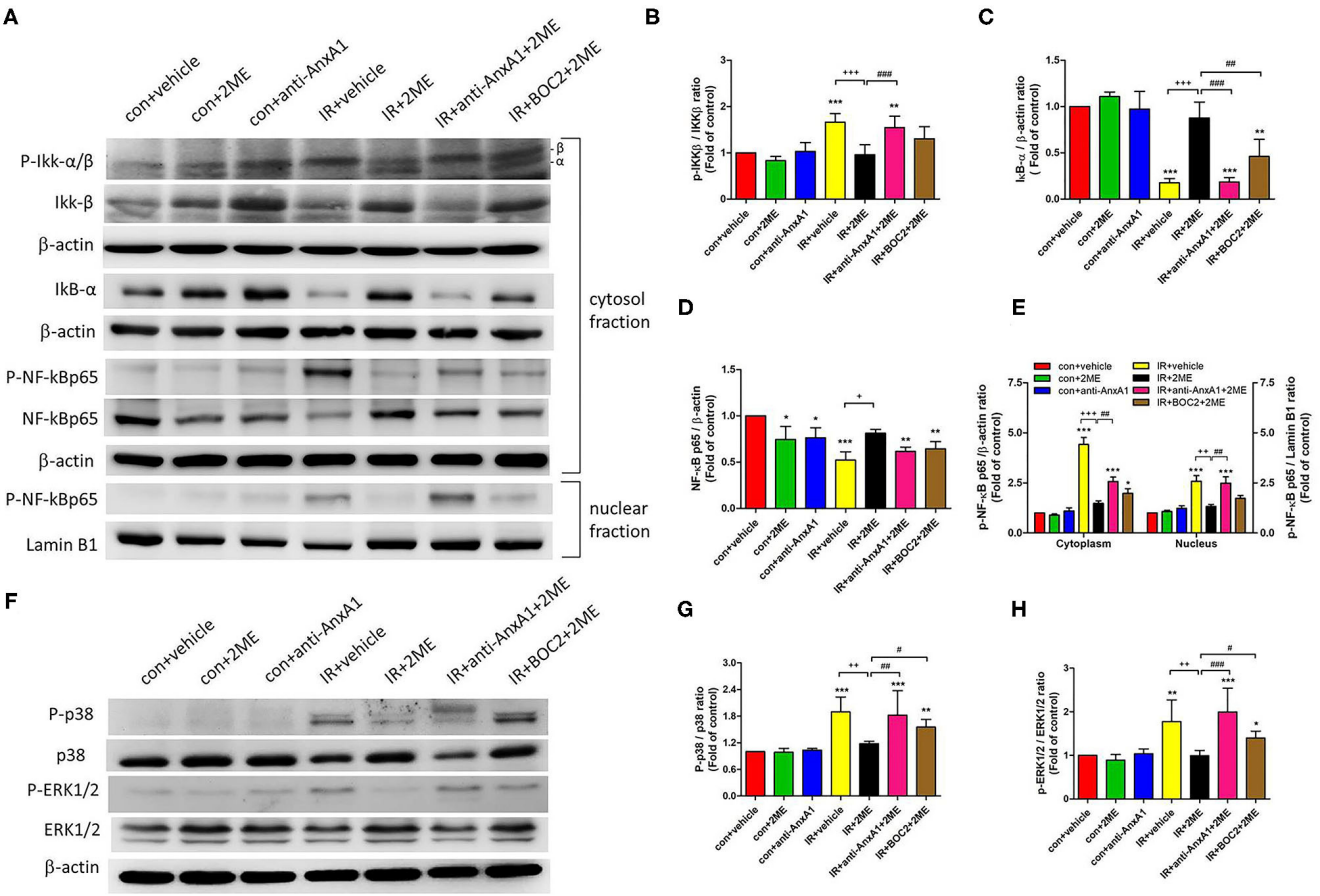
2ME repressed IR-induced NF-κB and MAPK activation in the lung tissue. **(A–E)** NF-κB and **(F–H)** MAPK signaling were detected in the lung tissue using Western blotting. 2ME reduced the **(B)** P-IKK-β, **(D,E)** nuclear and cytoplasmic P-NF-κB p65, **(G)** P-p38, and **(H)** P-ERK 1/2 levels, and increased the **(C)** IκB-α level in response to IR-induced lung injury. The addition of the anti-AnxA1 antibody or BOC2 significantly attenuated the inhibitory effects of 2ME on the NF-κB and MAPK signaling pathways. Lamin B1 and β-actin served as loading controls for nuclear and cytoplasmic proteins, respectively. ^*^p < 0.05, ^**^p < 0.01, and ^***^p < 0.001 compared with the control group; ^+^*p* < 0.05, ^++^*p* < 0.01, and ^+++^*p* < 0.001 compared with the IR group; and ^#^*p* < 0.05, ^##^*p* < 0.01, and ^###^*p* < 0.001 compared with the IR + 2ME group.

Compared to the vehicle control, IR injury significantly increased the phosphorylation of IKK-β, degradation of IκB-α in the cytoplasm, and nuclear translocation of NF-κB p65. Compared to rats subjected to IR injury, rats pretreated with 2ME prior to IR injury presented significantly decreased IKK-β phosphorylation, increased IκB-α levels, and the attenuated nuclear translocation of NF-κB p65 (Figures 7A–E). The levels of phosphorylated p38 and ERK were significantly increased by IR and reduced by the addition of 2ME compared to the vehicle control (Figures 7F–H). The administration of the anti-AnxA1 antibody or BOC2 attenuated these effects of 2ME.

### 2ME Upregulated AnxA1 Expression in HPAECs

**Figure 8:**
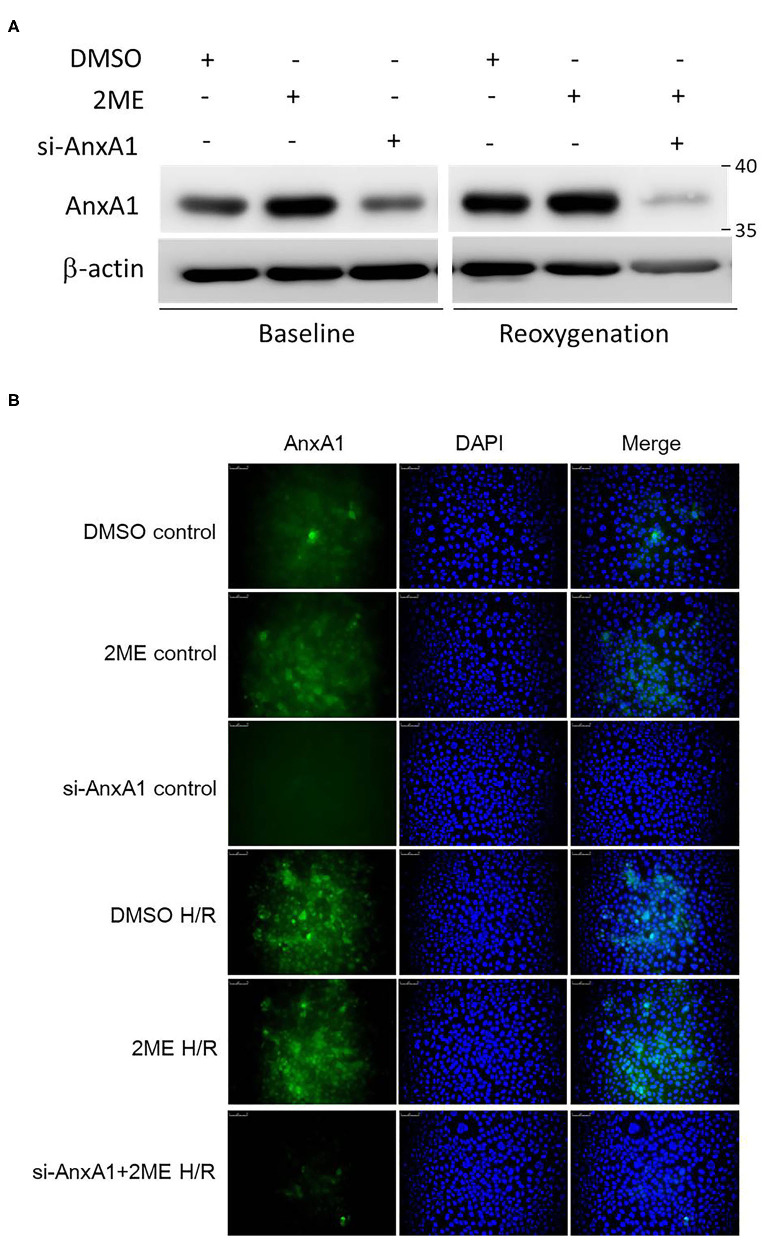
2ME increased AnxA1 expression in HPAECs. AnxA1 expression was detected using **(A)** Western blotting and **(B)** immunofluorescence staining. 2ME increased the expression level of AnxA1 in unstimulated cells. Compared to vehicle control, AnxA1 expression was substantially increased after HPAECs exposed to hypoxia for 24 h and reoxygenation for 1 h. No significant difference was observed between the HR and 2ME + HR groups.

We evaluated HPAECs using Western blotting (Figure 8A) and immunofluorescence staining (Figure 8B) to verify that AnxA1 was expressed in the lung. The viability of HPAECs was determined using WST-1 assays, and no cellular toxicity was detected when cells were treated with up to 1 μM 2ME (Supplementary Figure 5A). The level of the AnxA1 protein was obviously increased after pretreatment with 2ME at an optimal concentration of 1 μM under control condition (Supplementary Figure 5B). Compared with the vehicle control group, higher AnxA1 expression was detected in the HR group. However, no obvious differences in AnxA1 expression were observed between the HR group and the 2ME + HR group, despite the slight increase was observed after the 2ME pretreatment using Western blotting and immunofluorescence staining (Figures 8A,B).

### 2ME Attenuated NF-κB Activity and IL-8 Production and Increased ZO-1 Expression in HPAECs Subjected to HR

**Figure 9:**
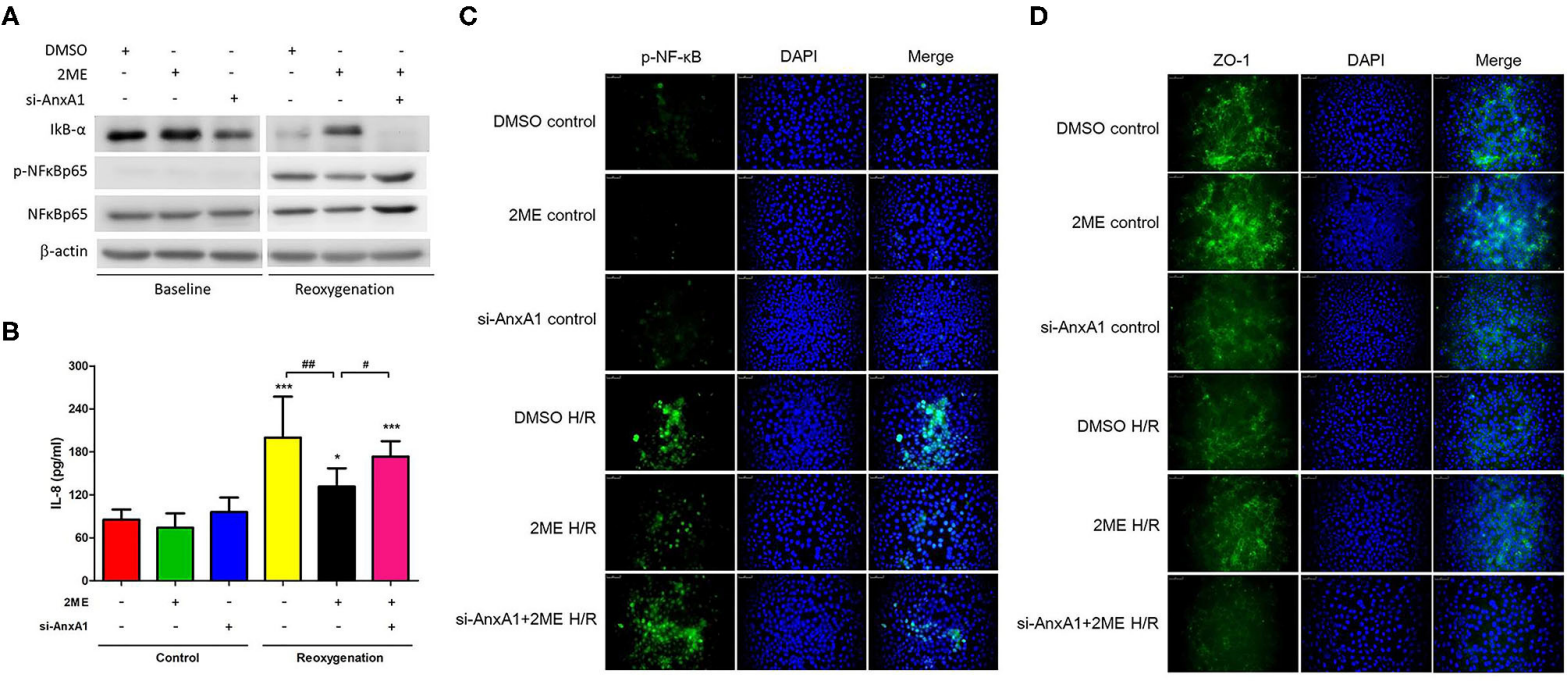
2ME suppressed NF-κB expression and IL-8 production, and enhanced ZO-1 activity in HPAECs upon HR injury. NF-κB and ZO-1 activity were detected using **(A)** Western blotting and/or **(C,D)** immunofluorescence staining. **(A,C)** 2ME increased the expression level of IκB-α, reduced the P-NF-κB and **(B)** IL-8 production, and increased **(D)** ZO-1 expression in HPAECs exposed to hypoxia for 24 h and reoxygenation for 1 h. Transfection with the AnxA1 siRNA reversed the 2ME-induced inhibition of NF-κB signaling, anti-inflammatory effects and increases in the levels of tight junction proteins in HPAECs. The results are representative of three independent experiments and presented as the means ± SD. ^*^p < 0.05 and ^***^p < 0.001 compared with the control group; and ^#^*p* < 0.05 and ^##^*p* < 0.01 compared with the HR + 2ME group.

We established HR models in HPAECs to determine whether 2ME exerted its protective functions *in vitro*. Compared with vehicle control, the induction of HR in HPAECs led to increased phosphorylation of NF-κB p65, decreased expression of IκB-α and ZO-1, and increased levels of IL-8. 2ME (1 μM) significantly suppressed all of these effects of HR. However, these protective effects of 2ME were abrogated by the AnxA1 siRNA (Figures 9A–D).

### 2ME Increased AnxA1 and Cleaved Caspase-3 Levels, and Attenuated the Transmigration and TNF-α Production in Rat Neutrophils Subjected to HR

**Figure 10:**
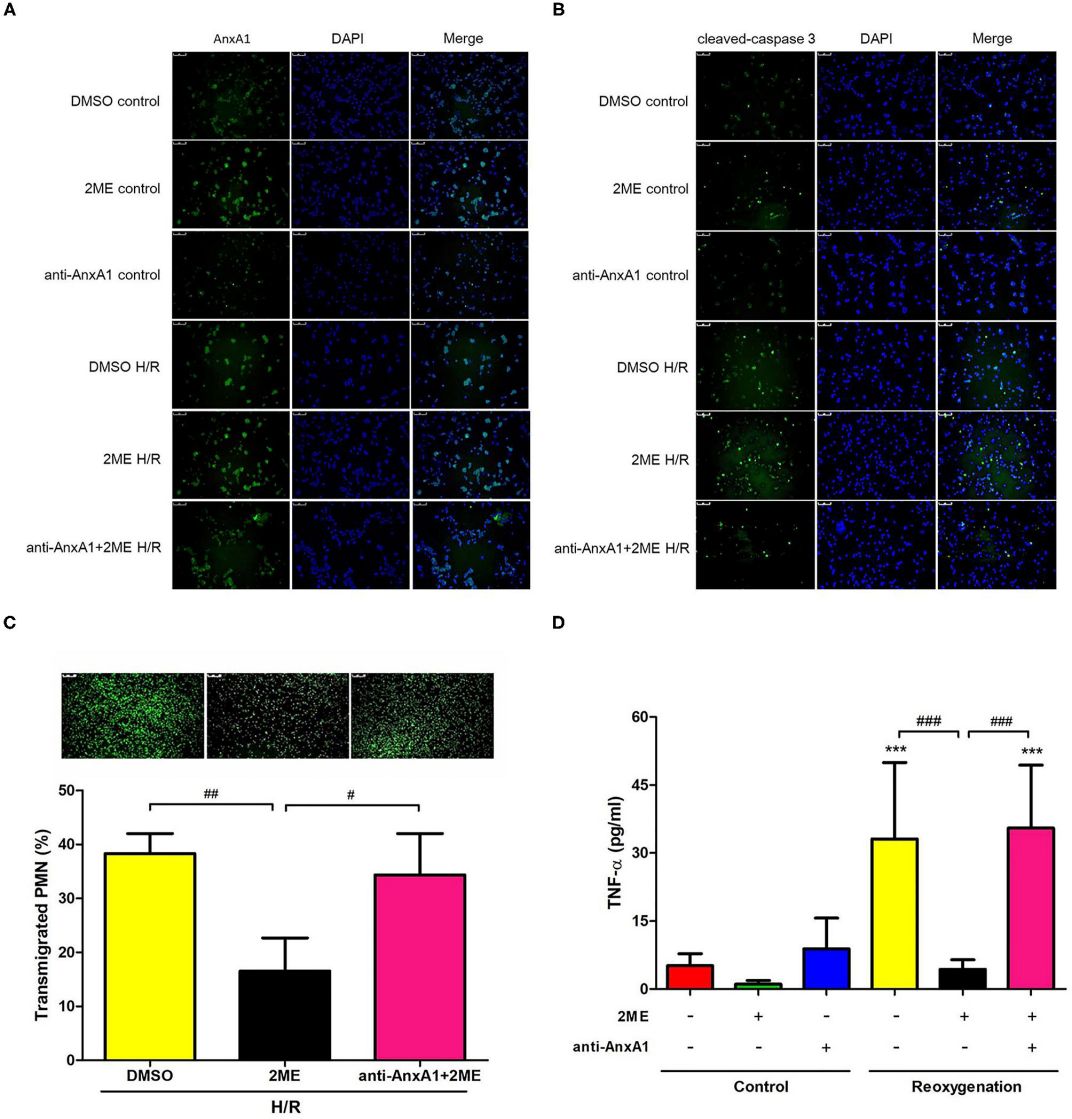
2ME increased AnxA1 and cleaved caspase-3 levels, and attenuated the transmigration and TNF-α production in rat neutrophils subjected to HR. AnxA1 and cleaved caspase-3 activity were evaluated using immunofluorescence staining **(A,B)**. Compared to the vehicle control group, 2ME increased AnxA1 and cleaved caspase-3 levels in the 2ME control group. Compared to the vehicle control group, the HR group presented higher AnxA1 and cleaved caspase-3 levels. 2ME administration further increased AnxA1 and cleaved caspase-3 levels in neutrophils exposed to HR. Rat neutrophils were stimulated with HR, induced to migrate across into the lower well of the migration chamber, and evaluated using a Leica DMi8 fluorescence microscope. 2ME attenuated **(C)** neutrophil transmigration and **(D)** TNF-α production after 2 h of hypoxia and 1 h of reoxygenation. These effects of 2ME were reversed by the anti-AnxA1 antibody. Normal mouse IgG was used as the control antibody in all experiments. ^***^p < 0.001 compared with the control group; ^#^*p* < 0.05, ^##^*p* < 0.01, and ^###^*p* < 0.001 compared with the HR + 2ME group.

Rat neutrophils were cultured with 2ME and then subjected to HR and pretreated with the anti-AnxA1 antibody at concentrations of 0.5, 1, and 2 μg to investigate the anti-inflammatory effects of 2ME. WST-1 assays revealed that no cellular toxicity was detected upon the addition of up to 2 μM 2ME (Supplementary Figure 5C). The 2ME (2 μM) pretreatment increased AnxA1, FPR1&2, and cleaved caspase-3 levels in the unstimulated neutrophils. Compared to the vehicle control, the neutrophils subjected to HR injury expressed AnxA1, FPR1&2, and cleaved caspase-3 at higher levels. Higher levels of AnxA1 and cleaved caspase-3, and lower expression of FPR1&2 were observed in the 2ME + HR group than in HR only group (Figures 10A,B and Supplementary Figures 7B,C, 8B,C). In addition, 2ME reduced HR-induced neutrophil transmigration in a dose-dependent manner and TNF-α production (Figures 10C,D and Supplementary Figure 6). However, these effects of 2ME on the neutrophils were suppressed by the pretreatment with the anti-AnxA1 antibody (Figures 10A,D and Supplementary Figure 6).

## Discussion

In the present study, 2ME significantly upregulated AnxA1 expression and attenuated IR-induced lung edema, neutrophil infiltration, inflammatory cytokine production, oxidative stress, lung cell apoptosis, tight junction protein disruption, MAPK activation, and nuclear translocation of NF-κB in the lung tissue. In addition, the 2ME pretreatment exerted similar anti-inflammatory effects on HPAECs exposed to HR. Moreover, 2ME increased neutrophil apoptosis and decreased HR-induced neutrophil transmigration and TNF-α production. Finally, these protective effects of 2ME were abrogated by the anti-AnxA1 antibody or BOC2 in the animal model, by the AnxA1 siRNA in HPAECs, and by the anti-AnxA1 antibody in rat neutrophils. Based on these findings, 2ME ameliorates IR-induced acute lung inflammation by activating AnxA1-mediated anti-inflammatory effects.

Accumulating evidence has revealed a crucial role for 2ME in the immunomodulation of inflammatory diseases. Many studies have shown that 2ME reduces antigen-induced airway inflammation (9, 25) and attenuates IgG immune complex-stimulated acute lung inflammation (26). In addition, 2ME decreases IR-induced hepatocellular damage (27), and ameliorates renal IR injury by inhibiting reactive oxygen species (ROS) and proinflammatory cytokine production (10). These studies suggest that 2ME may have potential value in protecting against lung IR injury. However, to date, no study has investigated the effect of 2ME on IR-induced acute lung inflammation, which is characterized by marked neutrophil infiltration in the lung interstitial and alveolar space. The migration of neutrophils from the pulmonary vasculature across the epithelium into the alveolar space has been shown to be a pathogenic factor during acute inflammatory lung injury (21, 28). As neutrophils are highly capable of producing ROS, they are responsible for a substantial proportion of the damage observed in IR-induced acute lung inflammation. In a previous study by Issekutz et al., 2ME significantly inhibited neutrophil migration to the joints in an adjuvant arthritis rat model (29). Here, we showed that 2ME suppressed IR-induced neutrophil infiltration and ameliorated oxidative damage in the lung tissue after a pretreatment. These observations were confirmed by the decreased numbers of MPO- and Ly6G-positive cells and decreased number of PMNs and MDA and protein carbonyl levels in the lung tissue.

Azevedo Loiola et al. have demonstrated that 17β-estradiol ameliorated LPS-induced brain inflammation through activation of AnxA1 (17). AnxA1 serves as an important anti-inflammatory regulatory factor in various animal models of inflammatory diseases, including multiple sclerosis, peritonitis, arthritis, and various types of IR injury (12, 16, 30). In addition, Purvis et al. reported that endogenous AnxA1 prevents insulin resistance and associated inflammatory complications in an experimental model type 2 diabetes (31). Under unstimulated conditions in the lung, the AnxA1 protein is localized to the cytoplasm of the epithelium, alveolar macrophages, and neutrophils (32). After exposure to various stimuli, the AnxA1 protein is trafficked to the plasma membrane and to be secreted, then it interacts with FPRs and mediates downstream cellular signaling in an autocrine and paracrine manner (33). FPRs are 7 transmembrane G-protein coupled receptors, and three subtypes have been identified in humans, including FPR1, FPR2, and FPR3. FPR1 is predominantly expressed in neutrophils and rapidly upregulated in response to various inflammatory stimuli, including sepsis and autoimmune diseases (34). In contrast to the specific anti-inflammatory effect of FPR1, FPR2 is capable of interacting with numerous ligands, resulting in either anti-inflammatory or pro-inflammatory effects, depending on different ligand-specific interactions (35). An FPR2 agonist has been reported to ameliorate hyperoxia-induced lung injury in mice (36). AnxA1 binds FPR1&2 and subsequently inhibits neutrophil transmigration, promotes neutrophil apoptosis, and decreases the inflammatory response (13, 37). Our group and other researchers have shown that BOC2 (the FPR pan antagonist) abrogates the protective effects of Ac2-26 (exogenous AnxA1 peptide) on various types of IR injury (13, 16). Therefore, in the current study, we pretreated rats with BOC2 prior to 2ME administration to assess whether the protective effect of 2ME would be attenuated by an AnxA1 receptor antagonist, indirectly confirming that the effect of 2ME was mediated by AnxA1 signaling.

We and other investigators have shown that AnxA1 expression is upregulated during IR injury and accompanied by neutrophil infiltration (16, 38). In our study, 2ME increased endogenous AnxA1 expression in the lung tissue, HPAECs, and neutrophils under unstimulated conditions. However, a decreased AnxA1 expression in rat lungs was observed in the 2ME + IR group which was different from the 2ME + HR group in HPAECs or neutrophils. No significant difference was observed between the HR and 2ME + HR groups in HPAECs or neutrophils, despite the slight increase was observed after the 2ME pretreatment. We suggested that 2ME reduced AnxA1 expression after IR injury potentially by suppressing the infiltration of inflammatory neutrophils following an IR insult. *In vivo*, 2ME reduced the substantial influx of neutrophils expressing high levels of AnxA1 into the IR-injured lung, as evidenced by AnxA1, MPO, and Ly6G immunohistochemistry, immunoblotting, and double immunofluorescence staining of the lung tissue. *In vitro*, 2ME significantly suppressed HR-induced neutrophil transmigration and TNF-α production in rat neutrophils and increased ZO-1 expression in HPAECs, consistent with the *in vivo* results. Moreover, the anti-AnxA1 antibody, BOC2, or AnxA1 siRNA abolished the effects of 2ME on ameliorating IR injury *in vivo* or *in vitro*. These results further confirmed that the 2ME-induced anti-inflammatory effects were mediated by increasing the activity of the AnxA1 signaling pathway. Taken together, our study suggests that upregulating AnxA1 expression is mainly responsible for the protective effect of 2ME on IR-induced acute lung inflammation.

NF-κB, a regulatory transcription factor, is responsible for inducing the production of various cytokines and chemokines during inflammation. Under non-inflammatory conditions, IκB-α sequesters NF-κB dimers in the cytoplasm, which suppresses NF-κB nuclear translocation. IKK-β is an upstream regulator of NF-κB that regulates NF-κB activity by inhibiting IκB-α phosphorylation and degradation. IR causes IKK-β phosphorylation, subsequent IκB-α degradation, and NF-κB activation, which results in the production of proinflammatory cytokines, such as TNF-α and CINC-1 (16, 39). These increases in proinflammatory cytokine production further stimulate neutrophil recruitment and increase tissue injury. Our results was consistent with Liu et al. study showing that Ac2-26 decreases IKK-β activity and reduces oxygen-glucose deprivation/reperfusion-induced proinflammatory cytokine production in microglia (40). AnxA1 has been shown to attenuate NF-κB activation and downstream proinflammatory cytokine production in acute lung inflammation (16). In addition, Yeh et al. found that 2ME reduced LPS-induced ALI by inhibiting NF-κB signaling (8). In the present study, 2ME effectively inhibited the activation of the NF-κB signaling pathway and proinflammatory cytokine production in the IR-injured lung and HPAECs exposed to HR, consistent with previous research (8, 26). Moreover, the anti-AnxA1 antibody, BOC2, or siRNA pretreatment abolished the inhibitory effect of 2ME on NF-κB. Therefore, the regulation of the NF-κB signaling pathway via the upregulation of AnxA1 might be a possible mechanism by which 2ME protects against acute lung inflammation induced by IR. However, further studies are necessary to clarify how AnxA1 inhibits NF-κB signaling.

Claudin-3 and occludin are tight junction proteins that form physical barriers restricting the diffusion of solutes through adjacent epithelial cell, and maintain the integrity of the permeability barrier of the alveolar wall (41, 42). ZO-1, a scaffolding protein, interacts with claudin-3 and occludin, which is critical for tight junction regulation (41, 42). Decreased levels of tight junction proteins in lung tissue are reliable indicators of the occurrence of ALI (43). IR injury may disrupt the integrity of the tight junction proteins and induce capillary leakage, which is a key component of the pathogenesis of pulmonary edema (28, 43). Further, exogenous recombinant AnxA1 upregulates occludin in brain endothelial cells and enhances tight junction formation to maintain blood-brain barrier integrity (33). The absence of AnxA1 disrupts occludin organization and increased blood-brain barrier permeability (33). As shown in our previous study, the expression levels of ZO-1, claudin-3, and occludin were significantly reduced upon IR injury but restored by an exogenous AnxA1 peptide (16). In the current study, 2ME significantly increased the integrity of these tight junction proteins in lung IR injury and HPAECs exposed to HR, but the effect was abolished by the anti-AnxA1 antibody or siRNA pretreatment. Therefore, the ability of 2ME to inhibit tight junction protein disruptions may be related to increased AnxA1 protein expression.

Significant increases in the apoptosis of lung epithelial and vascular endothelial cells are required for the development of acute lung inflammation (44). Bcl-2, an apoptotic regulator, possesses potent antiapoptotic activity to support the survival of cells and is implicated in the protective effect on IR injury (28, 44). Caspase-3 is a crucial effector caspase in the apoptotic pathway. Lung IR injury has been shown to decrease the levels of Bcl-2 and increase the levels of cleaved caspase-3 in the lung tissue to activate lung cell apoptosis (45, 46). Excessive lung cell apoptosis impairs the epithelial barrier and results in acute lung edema. Recent studies have reported that 2ME prevents apoptosis by increasing the expression of Bcl-2 in kidney IR injury (10) and by inhibiting the expression of caspase-3 in animal models of diabetic retinopathy and global ischemia (47, 48). In our study, 2ME significantly attenuated IR-induced apoptosis in the rat lung tissue, as evidenced by the decreased level of cleaved caspase-3 and increased level of Bcl-2 in the lung tissue. In addition, the antiapoptotic effect of 2ME was hampered by neutralizing AnxA1. Based on these findings, 2ME may increase AnxA1 expression to suppress IR-induced lung cell apoptosis.

Another interesting finding was that 2ME induced neutrophil apoptosis *in vitro*, as evidenced by the increase in cleaved caspase-3 levels, and this change was substantially attenuated in the presence of the AnxA1 antibody. Lung IR injury induces neutrophil infiltration into the alveoli and promotes high levels of inflammatory cytokine production (2, 16). Prolonged neutrophil survival may lead to an uncontrolled inflammatory response at local inflammatory sites, which can be opposed by pro-resolving mediators such as AnxA1 (49). Several investigations have revealed that AnxA1 promotes neutrophil apoptosis by activating caspase-3 to avoid the subsequent devastating inflammation (37, 50). Based on the results of the current study, 2ME may induce neutrophil apoptosis by upregulating AnxA1 expression following IR-induced lung injury.

2ME is an orally active and well-tolerated anticancer drug with low toxicity that has completed phase I and II clinical trials. We observed that 2ME reduced lung damage in rats exposed to IR injury, and the effects of 2ME on the resolution of IR-induced acute lung inflammation are associated with the modulation of AnxA1 expression in alveolar epithelial cells and neutrophils. However, our study has some limitations. First, sex-specific differences in survival have been reported in patients with ARDS (51). We only used male rats for the experiment which limits the opportunity to better understand the effect of 2ME in female animals. Second, Singh et al. demonstrated that 2ME protected against angiotensin II-induced hypertension in ovariectomized female mice (52). Whether pre- or post-menopausal status can alter the efficacy of 2ME in this experiment was not clear. Future studies specifically designed to answer these questions will be needed. Third, the isolated lung IR model with 40 min ischemia and 60 min reperfusion in our laboratory can produce significant lung injury (16, 53), however, this animal model may not completely translate to human in clinical condition according to the value of PaO_2_/FiO_2_ ratio in Berlin definition (54). It may be due to this reason that only the perfusate gas levels could be measured but a discrepancy between blood and perfusate gas levels may exist.

In conclusion, 2ME ameliorates IR-induced acute lung inflammation by upregulating the expression of endogenous AnxA1 in the lungs, alveolar epithelial cells, and neutrophils. Our study thus provides a molecular rationale for the use of 2ME as a possible treatment for IR-induced acute lung inflammation. Further studies of the potential clinical efficacy of 2ME are needed to determine whether it represents an attractive candidate as a drug that protects against pulmonary IR injury.

## Data Availability Statement

The original contributions presented in the study are included in the article/Supplementary Material, further inquiries can be directed to the corresponding author/s.

## Ethics Statement

This animal study was reviewed and approved by the Institutional Animal Care and Use Committee of the National Defense Medical Center (approval number: IACUC-15-077, 19-March-2015).

## Author Contributions

W-IL, S-HT, K-LH, and S-JC participated in the research design. W-IL, H-PP, and S-YW conducted the experiments. S-YW and W-IL performed data analysis. W-IL and S-JC contributed to the writing of the manuscript. All authors contributed to the article and approved the submitted version.

## Conflict of Interest

The authors declare that the research was conducted in the absence of any commercial or financial relationships that could be construed as a potential conflict of interest.

## Supplementary Material

Supplementary Figure 1 Experimental design. **(A)** Experimental protocol showing the duration and time course of IR. **(B)** The experiment was designed to explore the roles of 2ME-related anti-inflammatory effects in IR lung injury.

Supplementary Figure 2 Further study of the potential mechanisms of 2ME-mediated protection in **(A)** HPAECs or **(B)** rat neutrophils exposed to HR.

Supplementary Figure 3 2ME increased the expression of the AnxA1 mRNA and protein and ameliorated IR-induced acute lung edema in a dose-dependent manner. **(A)** Pretreatment with 2ME increased the expression of AnxA1 mRNA and protein compared to the vehicle control group. IR injury significantly increased the **(B)** lung weight gain, **(C)** K_f_, **(D)** LW/BW, **(E)** W/D weight ratios, and **(F)** protein concentration in the BALF. Moreover, a significant decrease in these parameters was observed in the groups pretreated with 10 and 20 mg/kg BW 2ME prior to IR injury in a dose-dependent manner. ^*^*p* < 0.05, ^**^*p* < 0.01, and ^***^*p* < 0.001 compared with the control group; ^+^*p* < 0.05, ^++^*p* < 0.01, and ^+++^*p* < 0.001 compared with the IR group.

Supplementary Figure 4 2ME decreased polymorphonuclear leukocyte (PMN) infiltration induced by IR injury in the lung tissue. Lung tissues from each experimental group were immunostained with CD45, Ly6C, Ly6G, and Gal-3 antibodies and analyzed under a light microscope (200 × magnification). The number of CD45-, Ly6C-, Ly6G-, and Gal-3-positive cells in the rat lung tissues were markedly increased after IR lung injury, particularly Ly6G-positive cells. Compared with IR injury alone, IR-challenged rats with 2-ME administration displayed a marked reduction in the infiltration of CD45-, Ly6C-, Ly6G-, and Gal-3-positive cells. The anti-inflammatory effects of 2ME were abolished by the addition of the anti-AnxA1 antibody.

Supplementary Figure 5 The optimal 2ME concentration for inducing AnxA1 protein expression without cytotoxicity was 1 or 2 μM in HPAECs or rat neutrophils, respectively. Different concentrations of 2ME (0, 0.1, 1, 10, and 100 μM) or (0, 0.5, 1, 2, and 5 μM) were added to HPAECs or rat neutrophils for 24 or 2 h, respectively, prior to the detection of cell viability with WST-1 to define the cytotoxic concentration of 2ME. **(A,C)** No cellular toxicity was detected upon the addition of up to 1 μM 2ME in HPAECs and 2 μM 2ME in rat neutrophils. **(B)** Western blot showing differences in levels of the AnxA1 protein in unstimulated HPAECs were obtained from Western blotting after pretreated with 2ME at concentrations of 0, 0.1, 1, 10, and 100 μM. The optimal concentration of 2ME that induced AnxA1 protein expression was determined to be 1 μM among all investigated 2ME concentrations. **(D)** 2ME increased AnxA1 expression in unstimulated rat neutrophils, as detected using immunofluorescence staining. ^*^*p* < 0.05 compared with control group.

Supplementary Figure 6 Rat neutrophils were cultured with 2ME (2 μM) and then subjected to HR and pretreated with the anti-AnxA1 antibody (Santa Cruz Biotechnology, sc-12740) at concentrations of 0.5, 1, and 2 μg. Besides, Ac2-26 (100 μg) was added to investigate the neutralizing effect of the anti-AnxA1 antibody. Compared to the vehicle + HR group, the number of transmigrated neutrophils was significantly reduced in the 2ME + HR group. The anti-inflammatory effects of 2ME were abrogated by the pretreatment with the anti-AnxA1 antibody in a dose-dependent manner. Compared to the vehicle + HR group, the level of neutrophil transmigration was similar to the Ac2-26 +anti-AnxA1 antibody (2 μg) + HR group. Compared to the Ac2-26 + anti-AnxA1 antibody (2 μg) + HR group, a lower level of neutrophil migration was observed in the Ac2-26 + anti-AnxA1 antibody (2 μg) + 2ME + HR group. These results indicated that the anti-AnxA1 antibody can neutralize the exogenous AnxA1 peptide and abrogated the anti-inflammatory effect of 2ME.

Supplementary Figure 7 2ME modulated FPR1 expression in the lung epithelium and neutrophils. FPR1 expression was examined using immunohistochemistry **(A)** in lung tissue and immunofluorescence staining **(C)** in neutrophils. **(B)** FPR1 transcript levels in the neutrophils were assessed using real-time quantitative PCR. Compared to the vehicle control, 2ME increased FPR1 expression in the lung epithelium and neutrophils. IR induced prominent epithelial FPR1 staining in rat lungs and HR induced a marked increase in FPR1 staining and mRNA levels in neutrophils. Compared to the IR or HR group, the 2ME pretreatment reduced FPR1 expression in lung tissue from the 2ME + IR group and in neutrophils from the 2ME + HR group.

Supplementary Figure 8 2ME modulated FPR2 expression in the lung epithelium and neutrophils. FPR2 expression was examined using immunohistochemistry **(A)** in lung tissue and immunofluorescence staining **(C)** in neutrophils. **(B)** FPR2 transcript levels in the neutrophils were assessed using real-time quantitative PCR. Compared to the vehicle control, 2ME increased FPR2 expression in the lung epithelium and neutrophils. IR induced prominent epithelial FPR2 staining in rat lungs and HR induced a marked increase in FPR2 staining and mRNA levels in neutrophils. Compared to the IR or HR group, the 2ME pretreatment reduced FPR2 expression in lung tissue from the 2ME + IR group and in neutrophils from the 2ME + HR group.
