# Supplementary material for: 2-Methoxyestradiol Protects Against Lung Ischemia/Reperfusion Injury by Upregulating Annexin A1 Protein Expression
Source: Front Immunol. 2021 Mar 16;12:596376. doi: 10.3389/fimmu.2021.596376 (PMC8007881; doi:10.3389/fimmu.2021.596376)
Supplement: Supplementary file 1 [file Table_1.DOCX]

| Variable | Control | IR | IR + 2ME | IR + 2ME + Anti-AnxA1 |
| --- | --- | --- | --- | --- |
| pH |  |  |  |  |
| Baseline | 7.27±0.02 | 7.27±0.07 | 7.27±0.03 | 7.27±0.03 |
| Final | 7.24±0.01 | 7.21±0.03 | 7.23±0.01 | 7.22±0.04 |
| PaCO_2_ |  |  |  |  |
| Baseline | 36.1±1.1 | 36.6±1.5 | 36.3±0.9 | 36.4±0.8 |
| Final | 36.4±0.7 | 37.8±1.6 | 36.9±0.9 | 37.6±1.7 |
| PaO_2_ |  |  |  |  |
| Baseline | 150.8±2.6 | 152.1±1.1 | 150.7±2.2 | 150.5±1.9 |
| Final | 149.9±1.9 | 142.6±5.2 | 147.3±2.8 | 145.8±2.6 |
| ∆( Final- Baseline) | -1.7±0.6 | -9.2±3.9^*^ | -2.8±3.9^#^ | -4.8±2.5 |

**Supplementary** **Table 1**. Data for the blood gas analysis.

Data was expressed as mean ± standard deviation. * p < 0.05 compared with the control group; # p < 0.05, compared with the IR group.
