# Supplementary figures and images for: 2-Methoxyestradiol Protects Against Lung Ischemia/Reperfusion Injury by Upregulating Annexin A1 Protein Expression
Source: Front Immunol. 2021 Mar 16;12:596376. doi: 10.3389/fimmu.2021.596376 (PMC8007881; doi:10.3389/fimmu.2021.596376)

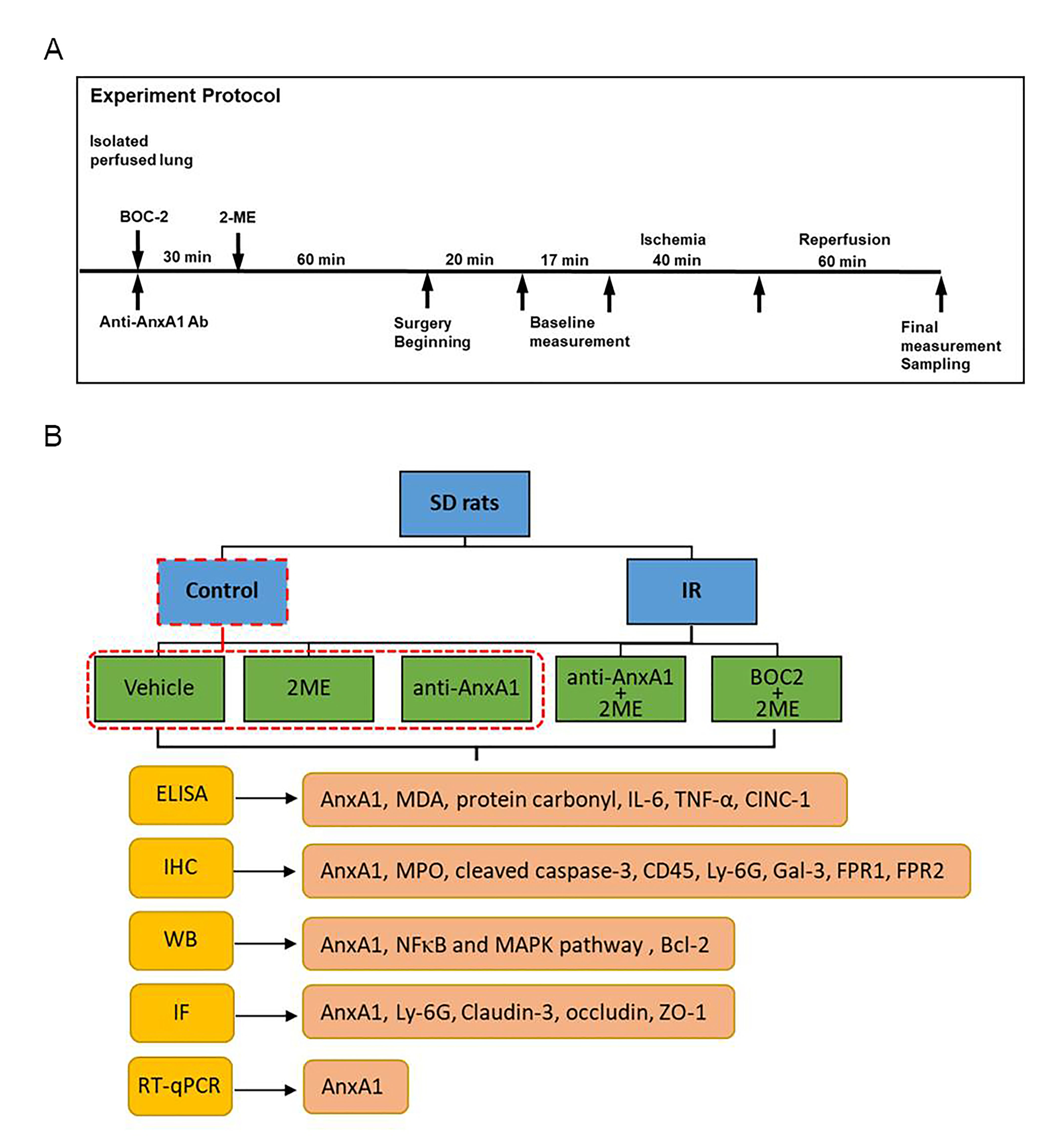

Supplement: Supplementary Figure 1 — Experimental design. (A) Experimental protocol showing the duration and time course of IR. (B) The experiment was designed to explore the roles of 2ME-related anti-inflammatory effects in IR lung injury. [file Image_1.JPEG]

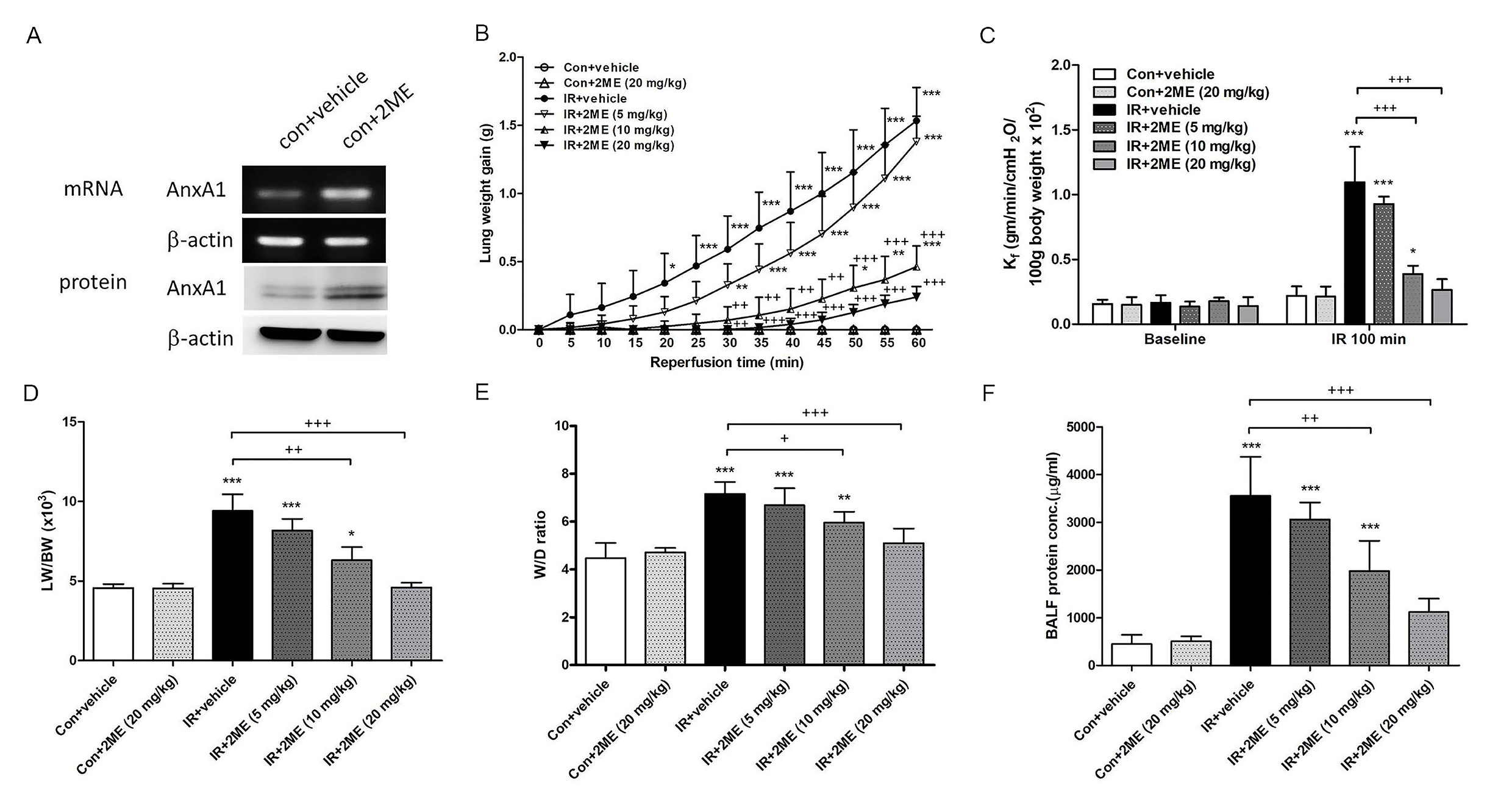

Supplement: Supplementary Figure 3 — 2ME increased the expression of the AnxA1 mRNA and protein and ameliorated IR-induced acute lung edema in a dose-dependent manner. (A) Pretreatment with 2ME increased the expression of AnxA1 mRNA and protein compared to the vehicle control group. IR injury significantly increased the (B) lung weight gain, (C) Kf, (D) LW/BW, (E) W/D weight ratios, and (F) protein concentration in the BALF. Moreover, a significant decrease in these parameters was observed in the groups pretreated with 10 and 20 mg/kg BW 2ME prior to IR injury in a dose-dependent manner. *p < 0.05, **p < 0.01, and ***p < 0.001 compared with the control group; +p < 0.05, ++p < 0.01, and +++p < 0.001 compared with the IR group. [file Image_3.TIF]

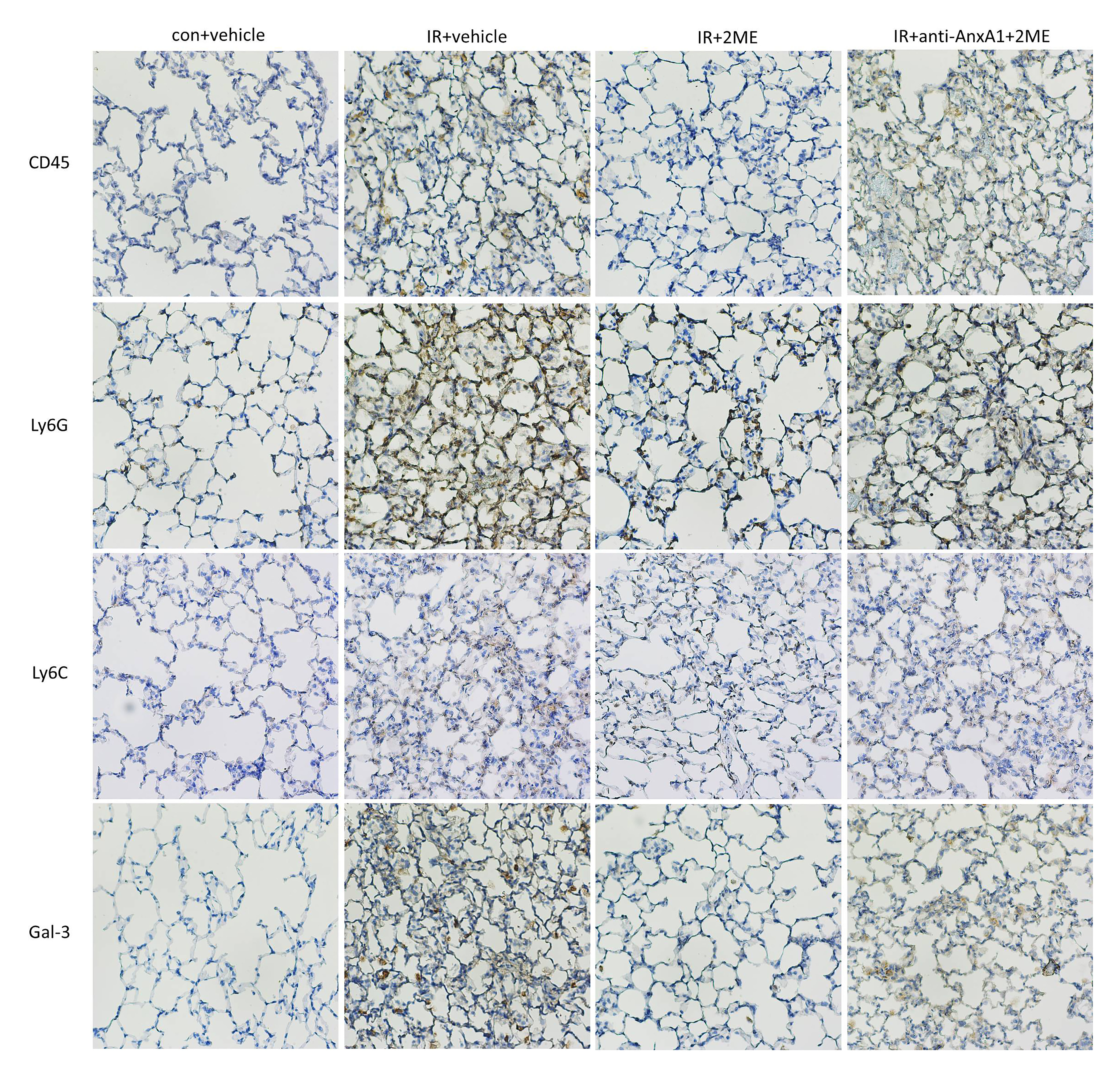

Supplement: Supplementary Figure 4 — 2ME decreased polymorphonuclear leukocyte (PMN) infiltration induced by IR injury in the lung tissue. Lung tissues from each experimental group were immunostained with CD45, Ly6C, Ly6G, and Gal-3 antibodies and analyzed under a light microscope (200 × magnification). The number of CD45-, Ly6C-, Ly6G-, and Gal-3-positive cells in the rat lung tissues were markedly increased after IR lung injury, particularly Ly6G-positive cells. Compared with IR injury alone, IR-challenged rats with 2-ME administration displayed a marked reduction in the infiltration of CD45-, Ly6C-, Ly6G-, and Gal-3-positive cells. The anti-inflammatory effects of 2ME were abolished by the addition of the anti-AnxA1 antibody. [file Image_4.JPEG]

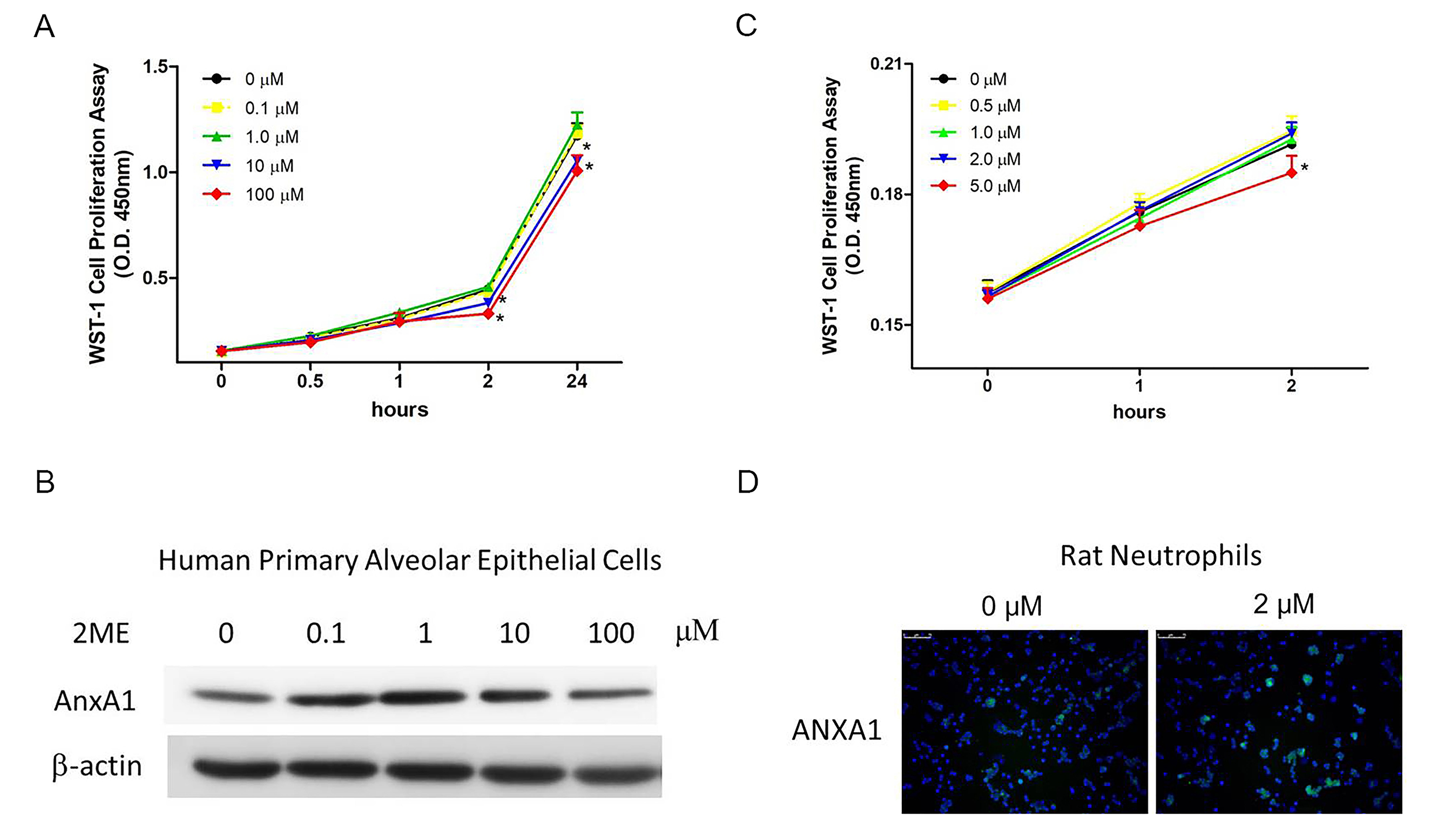

Supplement: Supplementary Figure 5 — The optimal 2ME concentration for inducing AnxA1 protein expression without cytotoxicity was 1 or 2 μM in HPAECs or rat neutrophils, respectively. Different concentrations of 2ME (0, 0.1, 1, 10, and 100 μM) or (0, 0.5, 1, 2, and 5 μM) were added to HPAECs or rat neutrophils for 24 or 2 h, respectively, prior to the detection of cell viability with WST-1 to define the cytotoxic concentration of 2ME. (A,C) No cellular toxicity was detected upon the addition of up to 1 μM 2ME in HPAECs and 2 μM 2ME in rat neutrophils. (B) Western blot showing differences in levels of the AnxA1 protein in unstimulated HPAECs were obtained from Western blotting after pretreated with 2ME at concentrations of 0, 0.1, 1, 10, and 100 μM. The optimal concentration of 2ME that induced AnxA1 protein expression was determined to be 1 μM among all investigated 2ME concentrations. (D) 2ME increased AnxA1 expression in unstimulated rat neutrophils, as detected using immunofluorescence staining. *p < 0.05 compared with control group. [file Image_5.TIF]

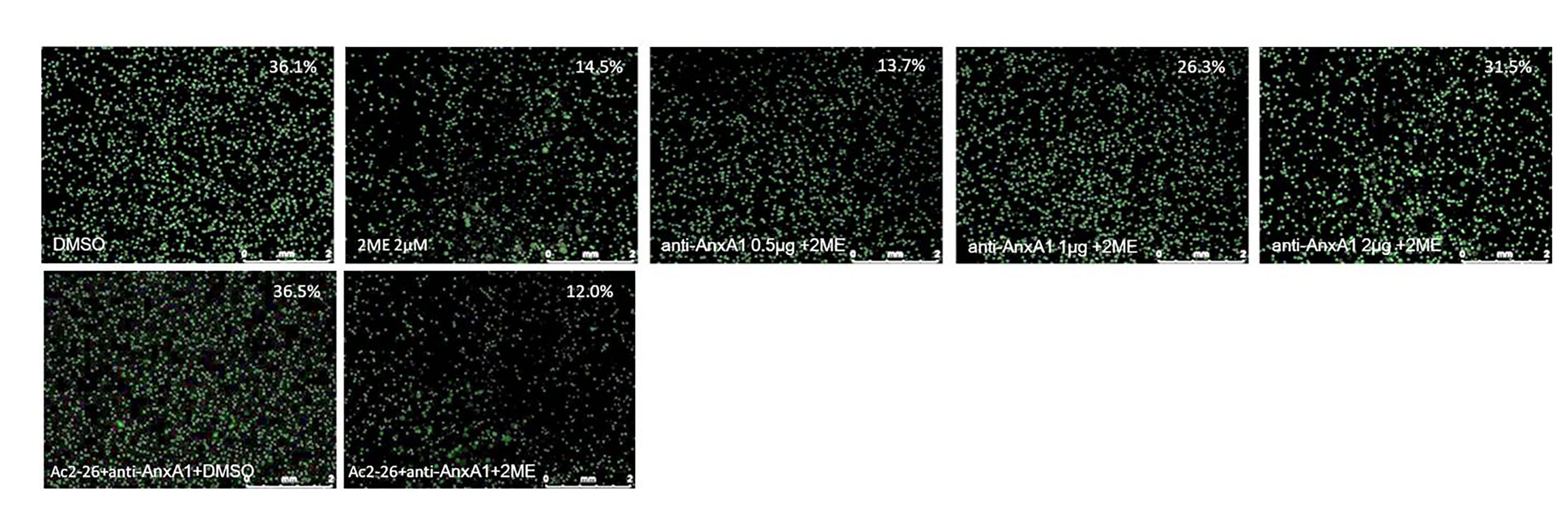

Supplement: Supplementary Figure 6 — Rat neutrophils were cultured with 2ME (2 μM) and then subjected to HR and pretreated with the anti-AnxA1 antibody (Santa Cruz Biotechnology, sc-12740) at concentrations of 0.5, 1, and 2 μg. Besides, Ac2-26 (100 μg) was added to investigate the neutralizing effect of the anti-AnxA1 antibody. Compared to the vehicle + HR group, the number of transmigrated neutrophils was significantly reduced in the 2ME + HR group. The anti-inflammatory effects of 2ME were abrogated by the pretreatment with the anti-AnxA1 antibody in a dose-dependent manner. Compared to the vehicle + HR group, the level of neutrophil transmigration was similar to the Ac2-26 +anti-AnxA1 antibody (2 μg) + HR group. Compared to the Ac2-26 + anti-AnxA1 antibody (2 μg) + HR group, a lower level of neutrophil migration was observed in the Ac2-26 + anti-AnxA1 antibody (2 μg) + 2ME + HR group. These results indicated that the anti-AnxA1 antibody can neutralize the exogenous AnxA1 peptide and abrogated the anti-inflammatory effect of 2ME. [file Image_6.JPEG]

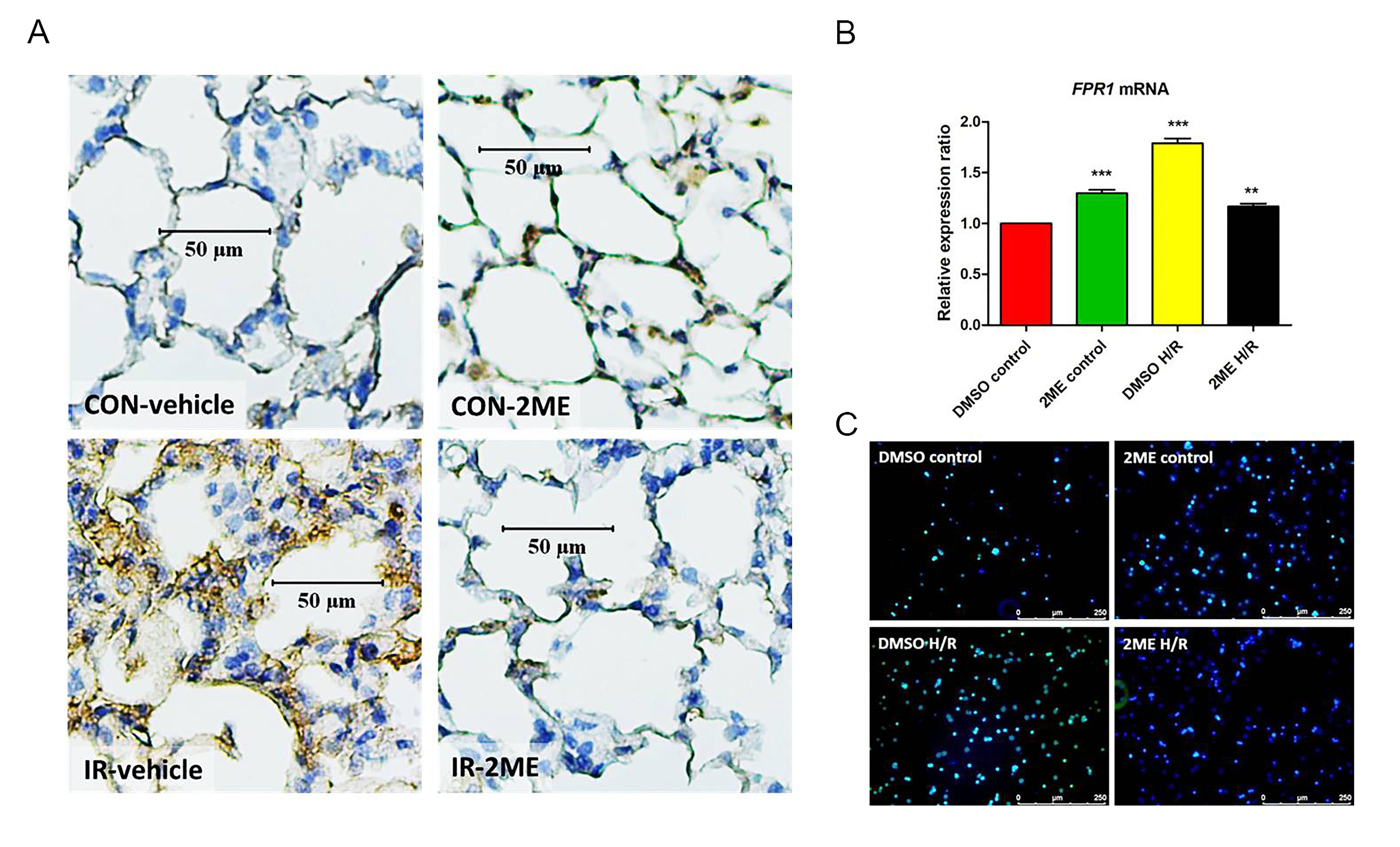

Supplement: Supplementary Figure 7 — 2ME modulated FPR1 expression in the lung epithelium and neutrophils. FPR1 expression was examined using immunohistochemistry (A) in lung tissue and immunofluorescence staining (C) in neutrophils. (B) FPR1 transcript levels in the neutrophils were assessed using real-time quantitative PCR. Compared to the vehicle control, 2ME increased FPR1 expression in the lung epithelium and neutrophils. IR induced prominent epithelial FPR1 staining in rat lungs and HR induced a marked increase in FPR1 staining and mRNA levels in neutrophils. Compared to the IR or HR group, the 2ME pretreatment reduced FPR1 expression in lung tissue from the 2ME + IR group and in neutrophils from the 2ME + HR group. [file Image_7.JPEG]

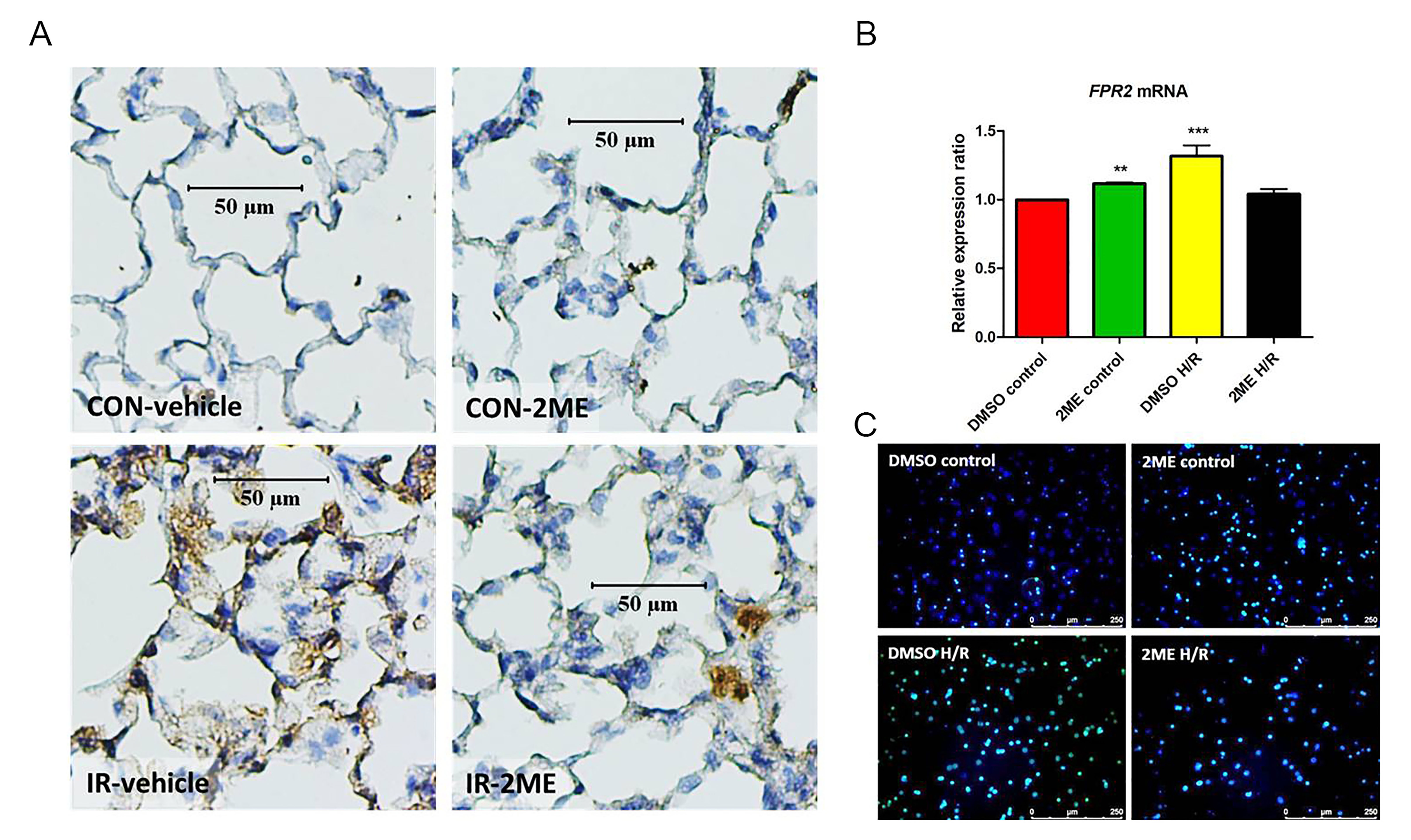

Supplement: Supplementary Figure 8 — 2ME modulated FPR2 expression in the lung epithelium and neutrophils. FPR2 expression was examined using immunohistochemistry (A) in lung tissue and immunofluorescence staining (C) in neutrophils. (B) FPR2 transcript levels in the neutrophils were assessed using real-time quantitative PCR. Compared to the vehicle control, 2ME increased FPR2 expression in the lung epithelium and neutrophils. IR induced prominent epithelial FPR2 staining in rat lungs and HR induced a marked increase in FPR2 staining and mRNA levels in neutrophils. Compared to the IR or HR group, the 2ME pretreatment reduced FPR2 expression in lung tissue from the 2ME + IR group and in neutrophils from the 2ME + HR group. [file Image_8.JPEG]
